# Source, co-occurrence, and prognostic value of *PTEN* mutations or loss in colorectal cancer

**DOI:** 10.1038/s41525-023-00384-7

**Published:** 2023-11-24

**Authors:** Ilya G. Serebriiskii, Valerii A. Pavlov, Grigorii V. Andrianov, Samuel Litwin, Stanley Basickes, Justin Y. Newberg, Garrett M. Frampton, Joshua E. Meyer, Erica A. Golemis

**Affiliations:** 1 Program in Cell Signaling and Microenvironment, Fox Chase Cancer Center, Philadelphia, PA 19111 USA; 2 Kazan Federal University, 420000 Kazan, Russian Federation; 3 Moscow Institute of Physics and Technology, 141701 Dolgoprudny, Moscow Region Russian Federation; 4 Biostatistics and Bioinformatics Facility, Fox Chase Cancer Center, Philadelphia, PA 19111 USA; 5 Greenfield Manufacturing, 9800 Bustleton Ave, Philadelphia, PA 19115 USA; 6 Foundation Medicine, Inc., 150 Second St., Cambridge, MA 02141 USA; 7 Department of Radiation Oncology, Fox Chase Cancer Center, Philadelphia, PA 19111 USA; 8 Department of Cancer and Cellular Biology, Lewis Katz School of Medicine at Temple University, Philadelphia, PA 19140 USA

**Keywords:** Molecular medicine, Colorectal cancer

## Abstract

Somatic *PTEN* mutations are common and have driver function in some cancer types. However, in colorectal cancers (CRCs), somatic *PTEN*-inactivating mutations occur at a low frequency (~8–9%), and whether these mutations are actively selected and promote tumor aggressiveness has been controversial. Analysis of genomic data from ~53,000 CRCs indicates that hotspot mutation patterns in *PTEN* partially reflect DNA-dependent selection pressures, but also suggests a strong selection pressure based on protein function. In microsatellite stable (MSS) tumors, *PTEN* alterations co-occur with mutations activating *BRAF* or *PI3K*, or with *TP53* deletions, but not in CRC with microsatellite instability (MSI). Unexpectedly, *PTEN* deletions are associated with poor survival in MSS CRC, whereas *PTEN* mutations are associated with improved survival in MSI CRC. These and other data suggest use of *PTEN* as a prognostic marker is valid in CRC, but such use must consider driver mutation landscape, tumor subtype, and category of *PTEN* alteration.

## Introduction

Colorectal cancer (CRC) is currently the third most common cause of cancer death in the United States, resulting in 53,000 deaths annually^1^, and is the second most common globally. Rates of some forms of colorectal cancers have been increasing for the past several decades^2,3^, including particularly in younger patients, with lifestyle factors one contributing factor^4^. The most common oncogenic drivers of CRC include loss of function (LoF) (by mutation or deletion) of the tumor suppressors *APC* and *TP53*, and activating mutations in *KRAS* or *BRAF*. Less common but occurring in ~8–9% of CRC are non-synonymous mutations in the tumor suppressor *PTEN*^5^, a lipid phosphatase that is a negative regulator of PI3K/AKT/mTOR signaling. The tumor suppressor role of PTEN is well-established in a number of cancers in which somatic *PTEN* mutations are very common, such as glioblastomas^6^. Cowden’s Syndrome and other syndromes associated with germline mutations impairing PTEN function are also associated with a high risk for several forms of cancer^7^. However, because of the lower mutational frequency, the prognostic and predictive role of *PTEN* mutations in affecting the biology and prognosis of CRC remains controversial. Particularly given the availability of drugs to target tumor-promoting signaling pathways negatively regulated by PTEN, an accurate understanding of how *PTEN* mutations arise, contribute to tumor presentation, and influence outcomes is of value.

In previous work, we analyzed mutational data from a large cohort of 34,129 CRC patients that allowed description of mutational patterns in *PTEN* and other genes associated with microsatellite stable (MSS) CRC, or CRC characterized by microsatellite instability (MSI)^5,8,9^. For *PTEN*, this revealed distinct patterns of mutational hotspots associated with MSS versus MSI tumors, with the MSS tumors more likely to be characterized by mutations that significantly impair the lipid phosphatase activity (LPA) of PTEN by destabilizing the protein and/or reducing protein enzymatic function, and correlation of specific mutational patterns with age of diagnosis with CRC^5^.

In this study, we have extended analysis to incorporate results from an additional 18,679 CRC tumor specimens, with many cases linked to data about prognosis. Initial analysis of the contribution of mutational signatures common in CRC to observed pattern of *PTEN* mutations indicated that although underlying mutational processes strongly influence the spectrum of observed *PTEN* mutations, other factors contribute to the selection process. One such factor is selection for impairment of PTEN function, as there is a strong bias for mutations causing PTEN destabilization or loss of lipid phosphatase activity. However, surprisingly, an analysis of the allele frequency of damaging versus non-damaging *PTEN* mutations showed both occurred at a similar frequency, arguing for additional selection exclusive of gene signature or impact on protein stability or lipid phosphatase activity. The correlation of *PTEN* mutations with specific driver mutations was observed, but varied with CRC subtype; for example, In MSS tumors, *PTEN* mutations had a marked pattern of co-occurrence with *BRAF* and *TP53* mutations that was not observed in MSI tumors. Intriguingly, the presence of *PTEN* alterations was prognostic, but in a nuanced manner, with PTEN deletions suggesting negative prognosis in MSS tumors, but *PTEN* mutations indicating positive prognosis in MSI tumors. These data suggest significant differences in the regulation and role of PTEN in MSS versus MSI CRC, and generally support a nuanced use of *PTEN* alterations as a prognostic factor.

## Results

### Profile of *PTEN* mutational hotspots

**Fig. 1:**
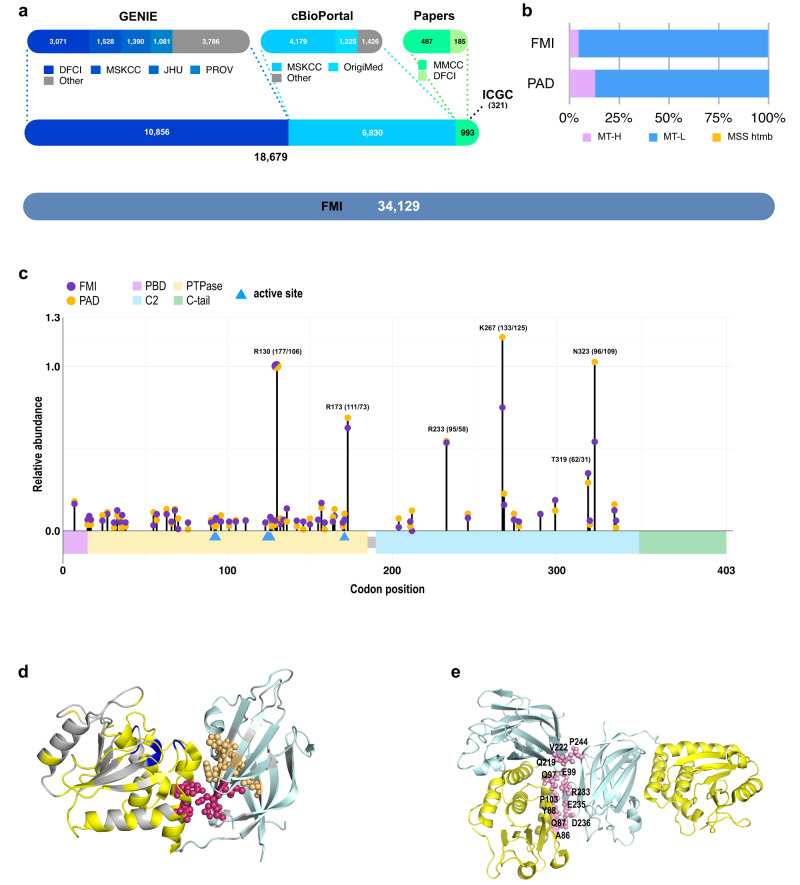
Description of the datasets used in the study, and identification of *PTEN* alterations and alteration hotsponts. **a** Publicly Available Dataset (PAD) for CRC used in this study, with information on numbers of CRCs acquired from AACR-GENIE, cBioportal, ICGC, and publications^10,58^; data were manually reviewed to avoid duplicated entry of tumors into the datasets, resulting in 18,679 non-redundant records. The FMI CRC dataset is shown for the comparison (not to scale). **b** Fractions of MT-L, MT-H, and MSS-htmb tumors in the PAD and FMI datasets. **c** Structural domains in PTEN include a phosphatidylinositol 4,5-bisphosphate (PIP2)-binding domain which regulates catalytic activity (PBD; residues 6–15, purple); a phosphatase domain active against phospholipids and phosphoproteins (residues 14–185, yellow; blue triangles mark active site residues); a C2 domain, which regulates PTEN localization (residues 190–350; light blue); and a C-terminal tail that influences protein stability and target specificity (residues 352–403; green). Location and relative abundance of *PTEN* hotspots in the PAD and FMI datasets. The height of each lollipop indicates the relative frequency of the corresponding mutation in the dataset, with the frequency of the most abundant observed hotspot in FMI set (R130) set as 1. For the top 6 hotspots, the absolute number of mutations in FMI/PAD datasets are shown in parentheses. Number of *PTEN* mutations analyzed: FMI - 2120; PAD- 1398. **d** PTEN 3D hotspots based on AlphaFold prediction. High-confidence (detected in all five top-ranking AlphaFold models with *p*-value < 0.005) novel 3D hotspots impacting the C2 domain and/or including residues at the phosphatase-C2 domain interface are shown in magenta (centered around D252) and light-orange (centered around F273). Other 3D hotspots (including those partially overlapping with the previously reported^5^) detected in at least one out of five best AlphaFold models with *p*-value < 0.005 are indicated in gray. These are predominantly located within the phosphatase domain. Phosphatase domain (left) is color-coded in yellow, and C2 domain (right) in blue. Specific affected residues are in Supplementary Table S3. **e** Location of PTEN residues at the dimer interface between two PTEN monomers, involving residues in the phosphatase (yellow) and C2 (blue) domains. See Supplementary Table S4 for specific affected residues.

We used an extended cohort combining colorectal tumor specimens from FMI, cBioportal, AACR-GENIE, and other sources of publicly available data (PAD) (Fig. 1a, Supplementary Fig. S1a, b), to augment and contrast with specimens available from FMI^5^. In the PAD dataset, 86.9% of tumors for which MS status was known or imputed were MSS/tumor mutation burden (TMB) low (designated MT-L), 13.0% MSI/TMB high (designated MT-H), and 0.1% MSS with a high TMB (MSS-htmb, often associated with *POLE* mutations) versus 94.6% MT-L, 4.7% MT-H, and 0.7% MSS-htmb in the FMI dataset (Fig. 1b; see discussion of rationale for use of MT-L, MT-H, and MSS-htmb terminology in methods and in^5^). The frequencies of *PTEN* alterations we observed in the combined PAD and FMI datasets are MT-L, 3.9%; MT-H, 18.1%; MSS-htmb, 44.0%.

Analysis of the pattern of recurrently mutated amino acids in *PTEN* (e.g. hotspots) indicated similar profiles in the FMI and PAD datasets (Fig. 1c, Supplementary Table S2). To determine if specific sites of mutation are clustered on the 3D folded structure of the protein, we modeled PTEN with Alphafold2 (AF2) and ESMfold (Supplementary Fig. S1c), which allowed us to extend the coverage beyond that present in the experimental models. Using the 5 best models (Supplementary Fig. S1d) we identified statistically significant 3D hotspots (Supplementary Table S3), defined as overabundance of mutations occurring in a group of amino acids no more than 5$\mathring{\rm A}$ apart in the PTEN monomer. This identified two new 3D hotspots partially encompassing the C2 domain of PTEN (Fig. 1d), in addition to 3D hotspots residing within the phosphatase domain. In addition, since PTEN is known to function as a multimer, we investigated whether mutations disrupt the PTEN dimeric interface. Interestingly, the interface between the PTEN molecules represents a “coldspot”, with mutations occurring there at ~3.8 times lower frequencies than elsewhere, in both MSS and MSI tumors (*p*-value = 0.003, Fig. 1e, see Supplementary Table S4 for specific affected residues).

### Overall selection for non-synonymous *PTEN* mutations

**Fig. 2:**
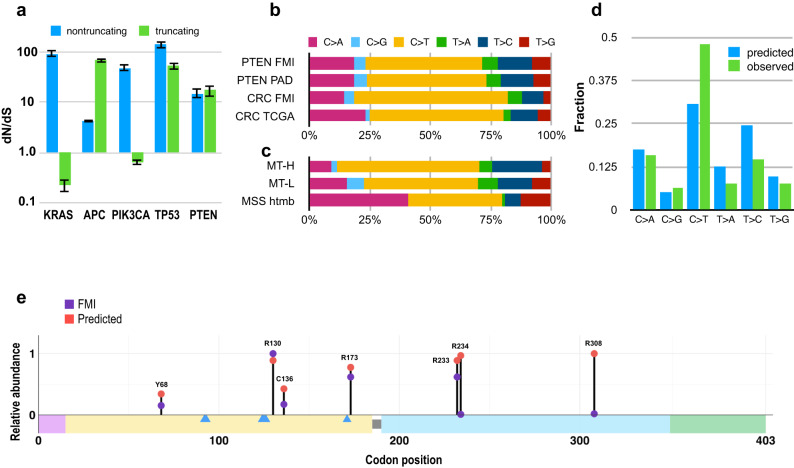
Relationship of selection pressures and *PTEN* mutation patterns. **a** Ratio of non-synonymous to synonymous (dN/dS) for non-truncating (blue) or truncating (green) mutations observed in tumors bearing mutations in *KRAS, APC, PIK3CA, TP53*, or *PTEN*. Error bars represent 95% confidence intervals. **b** Spectra of transitions and transversions for *PTEN* observed in the FMI and PAD subsets, compared to the frequency of these changes observed in all genes profiled by FMI in CRC, or previously published^67^ data on CRC specimens as baselines. **c** Spectra of *PTEN* transitions and transversions observed in the MT-L, MT-H and MSS-htmb subsets, based on specimens from the FMI dataset. **d** Frequency of observed (green) *PTEN* nucleotide changes in MT-L FMI subset versus values predicted based on nucleotide content (blue). The difference in the overall pattern of substitutions is statistically significant, *p*-value < 2.2e−16 (using a chi-square test). **e** Comparison of relative frequencies of top 5 predicted and top 5 observed single nucleotide substitution hotspots in the FMI MT-L subset. Frequency of the most abundant observed hotspot (R130) and the most abundant predicted hotspot (R308) was set as 1. Of the top 5 predicted hotspots, three (those in codons 130, 173, and 233) are also among the top 5 observed hotspots. Mutations in codons 234 and 308 are predicted to occur with high frequencies but have low counts in observed set. Observed hotspots in codons 136 and 68 occur at the locations predicted by the mutational spectra (albeit with much lower frequencies than those in codons 130, 173, and 233), but are not among the top 5 predicted hotspots. Supplementary Table S7 contains predicted and observed frequencies for all 403 codons of *PTEN*.

For most oncogenes and tumor suppressors with cancer driver activity, function is context dependent, with few universally important across a range of tissues and organs^10,11^. The driver status of *PTEN* mutations in CRC is ambiguous, with one study finding mutational inactivation of *PTEN* under selection in many tumor types, but not in CRC^12^. One approach to assess functional selection pressures in cancer genomes is to calculate the ratio of non-synonymous to synonymous mutations (dN/dS) in a tumor type-specific manner. Using this approach, *PTEN* mutations were identified as tumor drivers in 16/29 tumor types, including CRC^11^; however, this conclusion was drawn from the analysis of only 226 colorectal samples based on a complex statistical model. Using the extensive dataset in this study, we directly calculated dN/dS for *PTEN* benchmarked to the oncogenes *KRAS* and *PIK3CA*, and the tumor suppressors *APC* and *TP53*, considering non-truncating and truncating mutations separately (Fig. 2a). For *KRAS* and *PI3K*, there is a strong selection for non-truncating mutations and a negative selection against truncating mutations, reflecting the known requirement for specific missense mutations to activate these oncogenes. There is a very strong selection for *APC* truncating mutations, although non-truncating mutations are also positively selected. For both *PTEN* and *TP53* there is similarly a very strong selection for both truncating and missense mutations; although the dN/dS is smaller for *PTEN* (suggesting a slightly higher burden of passenger mutations), nevertheless about 94% of non-truncating and 95% of truncating *PTEN* mutations are expected to be genuine driver mutations. To gain additional insight, we analyzed additional features of *PTEN* mutational landscape, including the hotspot profile, allele frequencies, and co-occurrence profiles, and impact on survival.

### Nucleotide substitution profile in *PTEN* in CRC: mutational signatures versus functional selection

To investigate the underlying selection factors for *PTEN* hotspots, we first cross-validated the changes observed in the PAD and FMI datasets, comparing the specific nucleotide substitutions observed in *PTEN*, versus cumulative substitutions observed in all genes analyzed in CRC specimens in FMI or public data (Fig. 2b). This showed overall congruence between the two *PTEN* datasets. The observed differences, while statistically significant due to the very high sample count, likely reflect the distinct percentage of MT-L, MT-H, and MSS-htmb in the FMI versus PAD datasets, as these CRC subtypes have highly divergent nucleotide substitution profiles (Fig. 2c). We next used the frequencies of each possible base substitution in each of 96 different trinucleotide contexts (ref. ^13^, Supplementary Table S5) to predict the nucleotide substitution spectrum and frequency across the PTEN coding sequence in MT-L and MT-H tumors. The transitions/transversions profile actually observed in *PTEN* MT-L cohort versus predicted based on the nucleotide content of the gene was significantly different (Fig. 2d, Supplementary Table S6), suggesting the presence of additional mutability variables. We also analyzed in detail the changes from the most abundant classes of *PTEN* mutations produced by mutational signatures, common in CRC^14^, (the SBS1 and ID1/ID2/ID5/ID7 signatures (collectively designated hereafter as IDT, for total); Supplementary Fig. S2a–c). This indicated that deletions in polynucleotide runs are much more frequent than insertions in MT-H cohort, as is typical for cells with deficiency in mismatch repair (MMR) proteins^15^, while the opposite was true for the MT-L subset.

Focusing on non-synonymous single nucleotide substitutions in *PTEN* for the combined cohort (excluding MSS-htmb samples, comprising ~0.7% of the total, as too small a cohort to yield significant conclusions), we compared the observed frequency of mutations, versus the predicted frequency based on the occurrence of mutational signatures common in CRC (Supplementary Table S6). As illustrated in Fig. 2e, this baseline prediction model correctly identified many of the most frequent hotspots. Mismatches between predicted and observed hotspot frequencies are likely due to selection pressure at the protein level, and/or reflecting the influence of additional local factors such as nucleosome positioning or protein binding sites, all of which have been reported to influence background mutability^16^.

To discriminate functional selection for the effect of specific mutations on protein function from the mesoscale features of the nucleotide sequence, we therefore also compared the predicted versus observed frequency of synonymous mutations, as these are not expected to affect protein function (Supplementary Fig. S2, Supplementary Table S7) while sharing the same coarse features (such as replication timing and chromatin state) with non-synonymous mutations. This analysis identified statistically significant differences in patterns of occurrence from predicted frequency for synonymous mutations (*p*-value 0.0015, Supplementary Fig. S2), suggesting the presence of additional DNA-based mutational processes driving such deviations. However, the deviation from the predicted pattern of non-synonymous mutations (Supplementary Table S7) could not be explained by the same non-selective processes identified in the analysis of the synonymous mutations, as only ~1% of models generated distributions that have metrics similar to that of the data, implying additional selective pressures at the protein level, and/or localized effects involving specific potentially mutable codons.

### Loss of LPA activity and protein abundance contribute to selection for *PTEN* mutational hotspots

**Fig. 3:**
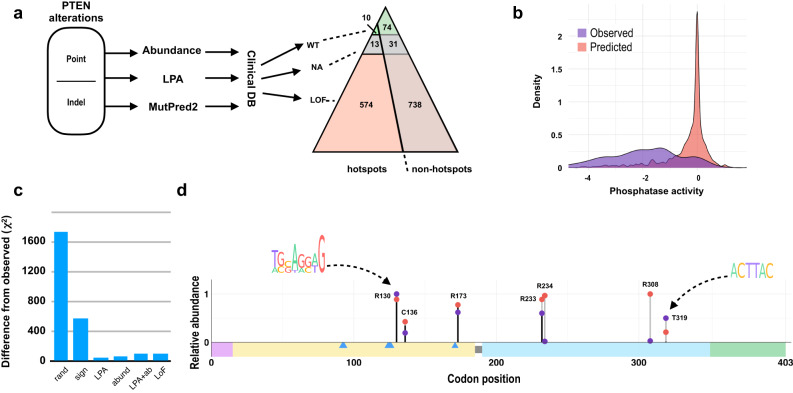
Functionality of mutationally altered PTEN at least partially defines the observed mutation spectrum. **a** For missense mutations in the MT-L dataset, analysis using predicted abundance and lipid phosphatase activity (LPA) based on^18^. Prediction of the impact of inframe deletions on protein function were done using MutPred2. After further stratification by annotation in the clinical databases (CKB, OncoKB, Clinvar; see Methods for details), hotspot versus non-hotspot mutations were assigned as functionally wild type (WT; green shading) or loss of function (LoF; pink shading); NA (gray), not available, signified insufficient data for prediction. Image depicts count of mutations in each functionally assigned subset of mutations (LoF, WT, NA) for both hotspots (left side of the triangle) vs non-hotspots (right side of the triangle). LoF mutations are more frequent among the hotspots (*p*-value < 2.2e−16). **b** Comparison of predicted (red) vs observed (blue) LPA profile for *PTEN* missense mutations in the MT-L subset. For each predicted or observed mutation, the corresponding LPA values were retrieved from ref. ^18^, see Supplementary Table S8. **c** Difference between predicted and observed mutational profiles using chi-square statistics. Rand, random baseline; sign, prediction made on the basis of mutational signatures alone; LPA, with correction for LPA score alone; abund, with correction for PTEN protein abundance score alone; LPA + ab, with correction for both LPA score and abundance; LoF, with correction for overall loss of activity, i.e., also taking into account annotations in clinical databases, where available. **d** Comparison of relative frequencies of top 5 predicted (based on mutational signature) and top 5 observed hotspots in the FMI MT-L subset, with the correction for LPA score. The height of the lollipop stems for the hotspots was calculated in relation to the frequency of the most abundant hotspot (R130 for the observed data, R308 for the predicted data), which were set as 1. For each codon, the total predicted value includes all possible mutations (in proportion determined by relative probabilities for each substitution, Supplementary Table S6); these mutations may or may not result in the loss of function. The overall height of the stem is color-coded to indicate the fraction of the total which would result in the loss of LPA (black), vs no loss of LPA (light-gray). Of the top 5 predicted hotspots, 3 (in codons 130, 173, and 233) are also among the top 5 observed hotspots. Different patterns of predicted versus observed characterize distinct hotspots, correlated with impact on protein function. For example, only about ~2/3 of mutations leading to amino acid changes in codon 233 are predicted to cause loss of LPA; here, the actual number of mutations is closer to the predicted count of mutations with low LPA scores. Mutations in codons 234 and 308 are predicted to occur with high frequencies based on mutational signature, but none of these is predicted to affect LPA; these positions are infrequently mutated in the analyzed cohort. Observed hotspots in codons 133 and 319 occur at the frequencies higher than that predicted by the mutational spectra; notably, these overlap with a consensus motif increasing the mutability of CG dinucleotides (ref. ^68^, left inset), or an indel hotspot consensus motif (ref. ^69^, right inset). Supplementary Table S9 contains predicted and observed frequencies for all 403 positions of PTEN, as well as the fraction of functionally damaging mutations according to LPA scores, abundance measures, and the combinations thereof.

Recently published estimates of the damaging potential of almost all possible *PTEN* mutations^17–19^, allowed us to characterize most of the observed single amino acid substitutions in PTEN in terms of protein abundance and/or lipid phosphatase activity (LPA) (Supplementary Fig S3a, Supplementary Table S8). Using these data, as well as computational tools to predict the impact of in frame deletions on PTEN protein function^20^ and annotations for specific *PTEN* mutations in databases linking genomic variation with phenotypes, we were able to estimate the damaging potential for >90% of non-synonymous mutations in *PTEN* (non-hypermutated subset), dichotomizing them as functional wildtype, versus LoF (Fig. 3a, Supplementary Fig S3b, Supplementary Table S8). This analysis indicated that hotspot mutations were significantly more likely to be annotated as LoF than non-hotspot mutations, in both the MT-L and MT-H subsets (*p*-value < 2.2e−16). In general, mutations with greater loss of LPA or abundance are observed at relative frequencies much higher than are those with less effect on protein function (Fig. 3b, Supplementary Fig. S3c), again implying the presence of positive selection for function-damaging *PTEN* mutations.

We therefore explored if adjusting for the functional status of *PTEN* mutations (applied as a coefficient calculated as fraction of function-damaging mutations in any given codon) would improve the ability to predict the hotspot profile. Correction for the LPA activity alone provided the best fit with the observed data, leading to a modest improvement in comparison with measures of protein abundance or overall LoF status; and resulted in the overall good congruency between predicted and observed data for single nucleotide substitution data (Fig. 3c, Supplementary Table S9). At least some of the remaining differences are likely due to fine scale mutational features, such as overlaps with mutation consensus sites, or sites with high DNA curvature (Fig. 3d insets, Supplementary Fig. S3d). Thus, as most critical selection processes, the profile of *PTEN* hotspots is first defined by mutational processes in CRC as defined by mutational signatures, and then further refined by secondary sequence features and positive selection for the loss of LPA.

### Evolutionary conservation and allele frequency of PTEN hotspot mutations suggests importance of non-canonical PTEN functions

**Fig. 4:**
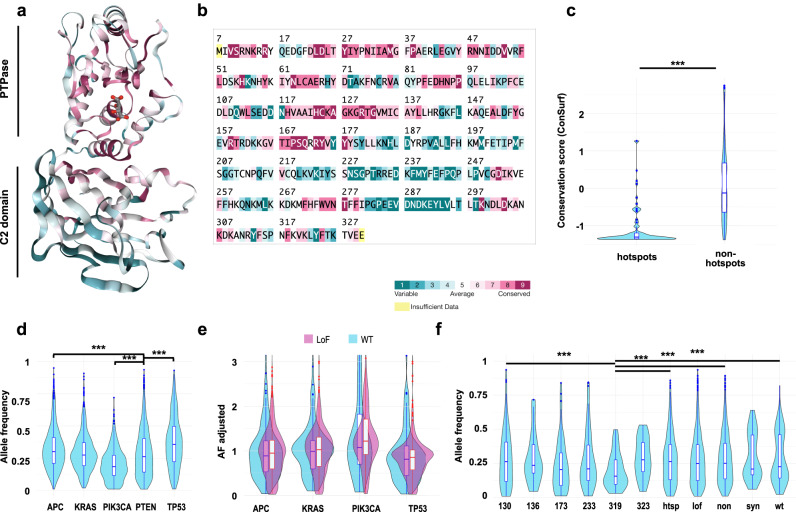
Evolutionary conservation, but not allele frequencies, differ between hotspot and non-hotspot mutations of PTEN. Structure of PTEN monomer (oriented with phosphatase domain to the top) (**a**) and primary amino acid sequence (**b**) color coded to indicate degree of conservation of residues. **c** Degree of conservation in mutations (missense and inframe indels) targeted by hotspot versus non-hotspot mutations indicates hotspot mutations target more highly conserved residues. Y-axis, Consurf score^70^ with the lower score indicating higher evolutionary constraint ***, *p* < 1.0E−12. **d** Allele Fraction (AF) of *PTEN* mutations, versus mutations in CRC driver genes, in MT-L CRC. **e** Allele frequency (AF) of *PTEN* mutations predicted as LoF (purple) or WT (blue) normalized to frequency of mutations in *APC*, *KRAS*, *PI3KCA*, or *TP53* in MT-L CRC. No statistically significant differences (all *p*-values > 0.05) were found between WT and LOF profiles, using a KS test. **f** Allele frequency of mutations in indicated hotspot residues, or cumulatively for mutations defined as “other hotspots” (htsp), LoF, non-synonymous (non), synonymous (syn), or WT. ***, *p* < 0.005. Lower AF of hotspot in codon 319 (which arises as a result of loss of one half of the repeat ACTTACTT and overlaps a consensus indel site, see Fig. 3d and Supplementary Fig. S2a) indicates it is a relatively late event in tumor progression.

Mutational hotspots overall are more frequent in evolutionarily conserved positions in protein-coding sequences, based on analysis of the TCGA Pancancer dataset^21^. We also analyzed the location of defined mutational hotspots in the context of the overall evolutionary conservation of both nucleotides and amino acid residues in *PTEN* (Fig. 4a–c). Across all CRC tumors, hotspot mutations were more likely than non-hotspots to occur in highly conserved amino acids of PTEN (mostly located in the phosphatase domain, Fig. 1c, Supplementary Fig. S3a). However, at the DNA level, hotspots did not occur in more highly conserved nucleotide positions (Supplementary Fig. S3e). These analyses suggested positive selective pressure for *PTEN* hotspot mutations at the level of protein function.

Driver mutations that alter protein activity typically occur earlier in tumor evolution, and thus have higher allele fraction (AF), in contrast to passenger mutations, which are expected to appear throughout cancer evolution and are frequently subclonal. For context, we compared the AF of *PTEN* mutations estimated to have LoF versus WT characteristics in tumors bearing *APC, KRAS, PIK3CA*, or *TP53* mutations (Fig. 4d) in the MT-L subset, comprising the bulk of CRC samples. Considering the complete set of *PTEN* mutations, the AF of *PTEN* mutations is lower than that of *APC* or *TP53* mutations, in agreement with the current view that alterations in *PTEN* are a later event in CRC carcinogenesis^22^; however, AFs are similar to those of *KRAS*, and higher than those of *PIK3CA*, suggesting selection. However, in the MT-L subset there was no significant difference in AF based on whether or not mutations impaired *PTEN* function (Fig. 4e). Further, comparison of the individual most abundant hotspots versus the set of all other hotspots, or mutations defined as LoF versus wt, or synonymous (Fig. 4f, Supplementary Table S10) in each case demonstrated a similar allele frequency in MT-L tumors. Comparison of AF of hotspots versus non-hotspots (Supplementary Fig. S3f, g) also demonstrated no significant differences. In contrast to earlier analysis (Fig. 3a–d), these data argued that any positive selection for function-damaging *PTEN* hotspot mutations is not reflected in their earlier appearance in the tumor evolutionary history.

Finally, we considered that mutations classified as wt-like based on LPA or protein stability may reflect disruption of alternative functions of PTEN, such as protein phosphatase activity, or interaction with substrate. Detailed analysis of the spatial distribution of these “wt” mutations of *PTEN* did not reveal any propensity to congregate in a particular PTEN domain, or in specific proximity to the sites of post-translational modifications (Supplementary Fig. S3h). Protein interactions have been reported for multiple domains of PTEN (Supplementary Fig. S3h); however, there is currently no available detail about whether the specific wt hotspot mutations would be critical mediators of such interactions.

### Distinct co-segregation of PTEN mutations in MT-L and MT-H tumors

**Fig. 5:**
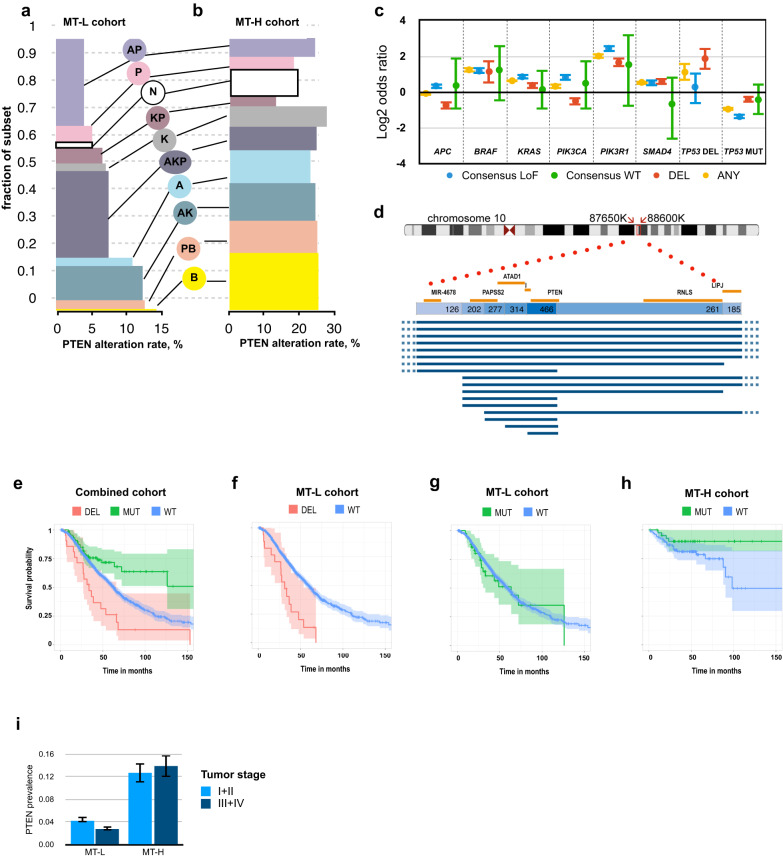
Correlation of *PTEN* alterations with alterations in other CRC driver genes, and with survival. *PTEN* alteration frequencies in the MT-L (**a**) and MT-H (**b**) cohorts, subdivided as in ref. ^10^ into functionally distinct groups according to the presence of mutations in *APC* (A), *TP53* (P), *BRAF* (B), *KRAS* (K), in combinations as indicated, or none (N) of these mutations. On the vertical axis, the height of each bar represents the fraction of MT-L tumors containing mutations in the indicated gene. On horizontal axis, fraction of non-synonymous *PTEN* alterations of any type. **c** Co-occurrence of PTEN LoF or WT-like mutations with alterations in *APC*, *TP53*, *PIK3CA*, *SMAD4*, *KRAS*, based on the merged FMI-PAD dataset for MT-L CRC. Error bars represent 95% confidence intervals. **d** Pattern of homozygous *PTEN* deletions in TCGA cohorts, summarized from all Pancancer studies. Schematic of the chromosome 10, coordinates 87650K-88600K, is shown. Top table, counts of all homozygous deletions in TCGA Pancancer set, encompassing *PTEN* and the genes indicated. Lines indicate the deletions encompassing *PTEN* specifically in the TCGA CRC cohort. **e** Analysis of survival in the complete AACR-GENIE Biopharma Collaborative Releases (BPC) CRC cohort, based on *PTEN* status. Red, homozygous *PTEN* deletions (*n* = 23); green, *PTEN* missense or indel mutations (*n* = 92); blue, *PTEN* wt (*n* = 1323). Shaded areas represent 95% confidence intervals. **f** Survival analysis for MT-L subset, homozygous *PTEN* deletion (red) vs PTEN wt (blue). Shaded areas represent 95% confidence intervals. **g** Survival analysis for MT-L subset, *PTEN* mutated vs *PTEN* wt. Shaded areas represent 95% confidence intervals. **h** Survival analysis for MT-H CRCs, *PTEN* mutated vs *PTEN* wt. Shaded areas represent 95% confidence intervals. Note a threshold of 0.005 is used in this study as a threshold for statistical significance (see Methods). **i** Frequencies of *PTEN* mutations by microsatellite stability cohorts and cancer stages (combined stages I & II, vs combined stages III & IV). Error bars represent 95% confidence intervals. See Supplementary Table S12 for details.

In complementary analysis, we asked if specific classes of *PTEN* alteration selectively co-occurred with common driver mutations that themselves were associated with significant survival effects (Fig. 5a, b). *APC, KRAS, TP53*, and *BRAF* mutations have markedly different distribution patterns in MT-L and MT-H cohorts, reflecting the distinct underlying genetic causes of these tumors, and distribution of these mutations has been used to develop a 10-group classification system associated with distinct clinical outcomes^10^. The frequency of *PTEN* alterations varied greatly between these groups in MT-L, but not MT-H tumors. In MT-L tumors, most *PTEN* alterations are observed in the highly prevalent groups of tumors characterized by mutations in *APC* + *KRAS*, *APC* + *TP53*, or *APC* + *KRAS* + *TP53*. However, the highest frequency of *PTEN* alterations is associated with tumors bearing *BRAF* mutations (*BRAF* & *BRAF* + *TP53* groups*)*, while the lowest frequency is observed in tumors bearing *APC and TP53* alterations in the absence of a *KRAS* or *BRAF* driver mutation. These patterns suggest biological selection for *PTEN* mutations in the context of specific patterns of driver mutations in the MT-L tumors. Intriguingly, more granular analysis of the relationship between *PTEN* and other driver mutations in MT-L tumors (Fig. 5c) indicates that the mutual exclusion between *PTEN* and *TP53* is driven entirely by the interaction of *TP53* mutations and *PTEN* LoF mutations; in contrast, *TP53* deletions tend to co-occur with *PTEN* deletions and other alterations. In contrast, with the exception of under-representation in tumors with *KRAS* + *TP53* alterations, similar frequencies of *PTEN* mutations are observed regardless of the profile of driver mutations in MT-H tumors.

PTEN is a negative regulator of PI3K, which is composed of a catalytic subunit, including PIK3CA, and negative regulatory subunits, including PIK3R1. In CRC, mutations activating PIK3CA or inactivating PIK3R1 occur at a low but appreciable frequency; these mutations significantly co-occurred with both LoF and WT mutations in *PTEN* (Fig. 5c). Similarly, *BRAF* and *KRAS* mutations significantly co-occurred with all classes of *PTEN* mutations. Additional analysis of the FMI and PAD subsets (Supplementary Fig. S4a, b), as well as analysis of the FMI subset for co-occurrence of mutations in key CRC driver genes, with either individual *PTEN* mutational hotspots, or cumulatively for belonging to other hotspots, or non-hotspots, also revealed no significant differences in co-occurrence frequency in MT-L (Supplementary Fig. S4c, d) or MT-H (Supplementary Fig. S4e) tumors. Considering co-occurrence separately for the different classes of LOF (e.g., by abundance or loss of LPA), in some cases there are differences (e.g., *BRAF*). This may reflect the importance of protein-protein interactions in which PTEN plays a non-catalytic function, which may be retained for PTEN mutant forms with reduced LPA, but reduced along with protein abundance.

### *PTEN* mutation versus deletion; relation to prognosis

Although most analyses of *PTEN* focus on mutation or epigenetic loss of expression of the gene, in some tumors, *PTEN* is only lost by deletion. Deletions of the *PTEN* gene were observed in the CRC data set, mostly in MT-L tumors, making up about 40% of all alterations (1228/3311 specimens with *PTEN* alterations in the combined FMI + PAD dataset). *PTEN* deletions most commonly co-occurred with the loss of the neighboring genes (Fig. 5d), as well as with the mutation of *BRAF*, *KRAS*, and/or *PIK3R*, and deletions in *TP53*, and were mutually exclusive with *TP53* mutations and *PIK3CA* mutations (Supplementary Table S11).

We evaluated the impact of *PTEN* mutation versus loss on overall survival of CRC patients (Fig. 5e). An initial analysis of pooled CRC specimens suggested opposing effects of non-synonymous *PTEN* mutations and *PTEN* deletions, with *PTEN* deletions associated with shorter survival (median overall survival (OS) 31.3 versus 63.2 months), but *PTEN* mutations with better survival (median OS not reached but >150 months). Independent analysis of the MT-L (Fig. 5f, g) and MT-H (Fig. 5h) cohorts confirmed that *PTEN* deletions were associated with worse outcome in MT-L patients (median OS 31.3 versus 60.0 months) (Fig. 5f). In contrast, non-synonymous mutations had no effect on survival in MT-L tumors (Fig. 5g), even though ~90% of them caused loss of PTEN LPA function. Intriguingly, a positive survival benefit of *PTEN* mutations was confined to MT-H tumors; however, this result did not reach statistical significance, likely due to inadequate sample size (Fig. 5h). This was unlikely to reflect specific cosegregation of *PTEN* mutations with specific driver mutations, which was not observed in MT-H tumors (Fig. 5b). TNM status could potentially influence outcome, if *PTEN* mutations were selectively associated with late-stage tumors. While stage information is not available for FMI specimens, we analyzed PAD data for 3221 MT-L samples and 796 MT-H samples (Fig. 5i, Supplementary Table S12). This indicated frequency of *PTEN* mutations was comparable in early (stage I and II) versus late (stage III and IV) tumors, excluding this interpretation.

In contrast, specific association of *PTEN* deletions with poor outcome in MT-L tumors may partially reflect the effect of alterations in other driver genes co-segregating with these deletions (Fig. 5a; e.g., mutation of either *BRAF*, *KRAS*, or *PIK3R1*, or deletion of *TP53* results in shorter survival (48 vs 68 months)). However, it may also reflect the loss of genes linked to *PTEN* in addition to or instead of *PTEN* itself. Analysis of the breakpoints of *PTEN*-encompassing deletions in CRC and other tumor types (Fig. 5d) indicated that genes commonly co-deleted with *PTEN* include *KLLN*, *ATAD1*, *PAPSS2, RNLS*, and *LIPJ*. Separate analysis of deletion patterns of these genes shows they are never in the absence of a co-occurring deletion of *PTEN*, and that mutations inactivating these genes in CRC are extremely uncommon, affecting <1% of tumors. However, it is possible that loss of one or more of these genes in combination with *PTEN* loss may worsen disease presentation; for example, loss of expression of *PAPSS2* has been linked to colitis and colon cancer^23^, and downregulation of *KLLN* exacerbates the presentation of Cowden syndrome^24^.

## Discussion

The predictive and prognostic value and functional role of *PTEN* alterations in various tumor types has long been an issue of clinical interest^25^. Although the relatively low frequency of genomic *PTEN* alterations in CRC has limited analysis of selection pressures in this tumor type, the large cohort analyzed in this study allows us to separate distinct contributions of mutational signature frequency and effect on protein function on the relative abundance of *PTEN* changes observed in MT-L and MT-H tumors, to establish co-segregation patterns with driver mutations, and to correlate *PTEN* alterations with overall survival. There are several key conclusions from these data. First, the prevalence of specific mutational signatures and other features of DNA strongly influence, but incompletely explain, the pattern of non-synonymous hotspot mutations observed in *PTEN*. Second, there is a strong selection for mutations that affect protein stability or lipid phosphatase activity of PTEN, independent of DNA signatures; related to this point, mutations are more likely to target highly conserved amino acids. Third, analysis of the allele frequency of *PTEN* mutations in MT-L tumors indicates that they occur as early in tumor evolution as do *KRAS* driver mutations, and earlier than *PI3KCA* mutations, suggesting that losing PTEN tumor suppressor activity may result in tumor development, at least in some cases. Opposing this idea, the fact that no significant difference is observed between AF of *PTEN* LoF mutations, versus those functionally wild type, arguing against functional selection based on lipid phosphatase activity or stability. Such functions might include control of PTEN intracellular localization^26^, protein phosphatase activity^27^, non-catalytic scaffolding activity^28^, or interaction with regulatory proteins^29^.

Fourth, *PTEN* mutations selectively occur within CRC subsets defined by the presence of specific driver mutations in MT-L tumors, including tumors driven by *BRAF*, *BRAF* + *TP53, APC* + *KRAS*, or *APC* mutations; in contrast, *PTEN* mutations are least likely to occur in tumors bearing *APC* + *TP53*, *TP53*, *KRAS* + *TP53*, *KRAS*, or *APC* + *KRAS* + *TP53* mutations (*P* < 2.2e−16), or lacking mutations in any of these genes. However, in many cases the frequency of cosegregation is similar whether the alteration in *PTEN* is predicted to cause LoF or not. In contrast, no specific pattern of co-occurrence is observed in MT-H tumors. Fifth, *PTEN* alterations appear to have prognostic value in an unselected set of CRCs, with deletions associated with worse outcome but mutations associated with an improved outcome. However, more detailed analysis of MT-L tumors indicates that mutations of *PTEN* have no prognostic value, whereas deletions, comprising about 40% of all *PTEN* alterations, are associated with worse outcomes that may reflect the biological function of loss of the PTEN protein or alternatively, may reflect the tendency of these deletions to co-segregate with driver mutations that are themselves associated with worse prognosis. Finally, in an intriguing finding, analysis of MT-H tumors suggests that in this cohort, *PTEN* mutations may be associated with greatly improved survival.

Interpretation of these findings is complicated, particularly as such interpretation informs the potential use of *PTEN* mutations for clinical prognosis. However, overall, several observations argue in favor of the idea that loss of PTEN activity promotes tumor growth in some but not all contexts of driver mutations. Most convincing among these is the fact that the majority of *PTEN* hotspot mutations (including 3D hotspots) are associated with loss of LPA function, in both MT-L and MT-H tumors. Also suggestive is the specific pattern of segregation observed in MT-L tumors, which reveals a strong bias towards selection of *PTEN* alterations in tumors bearing *BRAF* mutations. *KRAS* is the most common driver oncogene in CRC and many other tumors, based in large part on its ability to simultaneously activate *BRAF*, *PI3K*, and other signaling effectors, which collaborate to induce tumor proliferation, survival, and other processes associated with progression^30^. The fact that loss of PTEN, which would activate the PI3K effector pathway, specifically co-occurs with *BRAF* rather than *KRAS* mutation is compatible with the strong selection for activation of both RAS effector pathways. Notably, in all cases, the error bars for correlation with *PTEN* alterations deemed wild type in phenotype are considerably larger than those for LoF *PTEN* mutations. While this may partially reflect the lower abundance of these mutations, it may also reflect more uncertainty in calling the functional consequences of these mutations. For instance, these mutations may lead to the altered interactions between PTEN and protein partners; notably, there are >840 potential PTEN interactors listed in the BioGrid database (https://thebiogrid.org/111700/summary/homo-sapiens/pten.html), of which ~50 have so far been validated and are high confidence^31^.

The observed mutual exclusion of *TP53* and *PTEN* mutations is also interesting. TP53 is a transcriptional activator of *PTEN*^32^; conversely, PTEN blocks activation of MDM2, a ubiquitinating enzyme that inhibits TP53, and PTEN directly binds TP53 and regulates its transcriptional activity^33,34^. Mutual exclusivity of *PTEN* and *TP53* mutations has previously been documented in some breast cancers^35^, although the underlying mechanism has not been explored, and in some cancer types, dual loss of PTEN and TP53 contributes to robust tumorigenesis^36^. However, the fact that function-altering mutations rather than deletions in *TP53* and *PTEN* are mutually exclusive clearly implies altered activity of the proteins may contribute to the exclusion phenotype in CRC. Further analysis investigating PTEN functional interactions with *TP53* and other driver mutations, and incorporating transcriptional data, would be valuable as data becomes available. The lack of transcriptional data hinders comparison of the results of this study with other classification systems, such as the Consensus Molecular Subtypes (CMS) from the Colorectal Cancer Subtyping Consortium^37^, which depend heavily on use of transcriptomic profiling; conversely, only 2 of 18 datasets used to derive CMS have data on *PTEN* mutations. However, CMS1, comprising most of the MSI/MT-H samples, shows a tendency for higher *PTEN* mutations frequency (as well as for *BRAF* and *KRAS* mutations), in good concordance with our data. CMS3 appears to have a significantly lower frequency of *PTEN* deletions^37^. Direct study of these trends in an extended dataset is required.

A limitation of the current study is the lack of information for PTEN mRNA or protein levels in the FMI dataset, particularly as loss of *PTEN* expression in tumors is known to be induced by various forms of epigenetic regulation, including abundance of specific transcription factors that induce its expression, promoter hypermethylation, regulatory microRNAs, *PTEN* mRNA binding proteins, expression of the *PTENP1* pseudogene^38–42^. PTEN activity is further modified by a number of post-translational modifications that limit or enhance its activity^43^. To date, few studies have addressed the degree to which these mechanisms are pertinent in CRC; it is plausible that the contribution of reducing PTEN activity to CRC carcinogenesis is significantly underestimated at present.

Overall, the data in this study suggest that *PTEN* mutational status can have prognostic value in some CRC tumors, particularly in the set of MT-L tumors with *PTEN* deletions, and strikingly, for MT-H tumors with *PTEN* mutations. However, the basis for this prognostic value at present remains unclear, and for the MT-L tumors may well reflect the correlated deletion of *PTEN* with specific driver mutations that themselves are associated with poor prognosis. One question emerging from this analysis is whether the difference in prognosis associated with deletion rather than mutation of *PTEN* on prognosis reflects a property intrinsic to *PTEN*, or rather involves the loss of genes adjacent to *PTEN* on the chromosome, as deletions of *PTEN* typically involve simultaneous loss of *KLLN*, *ATAD1*, and *PAPSS2*. *KLLN*, encoding killin, is frequently lost together with *PTEN* in Cowden’s syndrome^44^, and contributes to DNA damage-induced apoptosis^44^ and control of genome stability^45^. *ATAD1* (ATPase family AAA domain-containing 1) is a member of a small family of proteins that influence mitochondrial function and cellular stress response, some of which have been linked to a role in cancer progression^46^; The loss of *ATAD1* predisposes cancer cells to apoptosis triggered by proteasome dysfunction, and alters survival in some cancer types^47^. *PAPSS2* encodes an enzyme that mediates the sulfation of numerous cellular proteins, including the mucins that form a protective layer of the intestinal epithelium; loss of *PAPSS2* has been shown to promote colitis and colon cancer^23^. The contribution of loss or epigenetic silencing of these *PTEN*-associated genes to the presentation of colorectal cancer has not to our knowledge been addressed.

A confounding factor to assessing the functional role of *PTEN* mutations in prognosis is the changing landscape of treatments for CRC over the past two decades, with initial dependence on cytotoxic chemotherapies augmented with use of antibodies targeting EGFR (typically for tumors lacking strongly activating *KRAS* mutations) regorafenib, and tiparacil/trifluidine. More recent investigations have focused on immunotherapies (for MSI-H tumors), and clinical trials are assessing additional protein-targeted inhibitors, including PI3K inhibitors. At present, although treatment information for large cohorts receiving a single treatment regimen is limited, there have been some studies investigating the impact of *PTEN* mutations or loss on response to these therapies. Given the importance of PI3K as a signaling effector for EGFR, a number of studies have implicated loss of PTEN in resistance to the EGFR-targeting antibodies cetuximab and panitumumab^48^. Unsurprisingly, both PTEN loss and activation of PI3K have been linked to resistance to PI3K-targeting drugs^49,50^. The data in this study emphasize some of the complexity of interpreting PTEN data in the context of resistance: for example, *PTEN* mutations might exert a dominant-negative effect over the wild-type protein, leading to AKT pathway activation; catalytically inactive PTEN might retain lipid phosphatase-independent activities such as promoting DNA repair that are lost after *PTEN* deletion. Such differences may have substantial effects on prognosis for survival after treatment.

Some work has linked *PTEN* loss to an immunosuppressive tumor microenvironment in various tumors^51–53^, which is likely to be an important consideration and topic for future mechanistic studies, given the rise in this class of agent. Although revolutionary in clinical cancer care, significant numbers of patients do not respond to immunotherapy. Understanding why these tumors are refractory to immunotherapy will help to improve the efficacy of treatment. Further, analyzing genes and proteins in both immune-sensitive and immune-resistant settings for differences in structure and function may provide further clues about how to predict and elicit responses. The data in this study emphasizes that it will be important to consider the potential predictive and prognostic value of PTEN loss with some degree of nuance, considering class of mutation, tumor subclass, and co-mutation spectrum.

## Methods

### Datasets analyzed

Comprehensive genomic profiling (CGP) was performed using the FoundationOne^®^ or FoundationOne®CDx assays (Foundation Medicine, Inc, Cambridge, MA, USA), as previously described^54^, using specimens collected from 2015–2019. Comparison data sets for studies with information on *PTEN* mutation status, and sex, age, and tumor subsite were collected from the cBioPortal for Cancer Genomics, https://www.cbioportal.org; AACR Project GENIE, https://genie.cbioportal.org^55^; the Catalogue Of Somatic Mutations In Cancer (COSMIC), https://cancer.sanger.ac.uk/cosmic^56^; ICGC, https://dcc.icgc.org^57^; and from data included in the supplementary tables of^10,58^. To remove redundancy of the collected data, we implemented the following prioritization rules: 1) All GENIE v.13 samples which overlapped with the studies in cBioPortal were removed. 2) For samples with outcomes progressively described in multiple publications, the studies reported in cBioPortal as of 01.01.2023 were used to establish survival data. 3) For samples without microsatellite (MS) status, but with measures of MS instability predicted by MSI prediction algorithms (e.g., SENSOR^59^ or MANTIS^60^ scores), we assigned MS status according to the cutoffs used in the corresponding studies (3.5 and 0.4, respectively; see below). 4) In cases where multiple specimens were collected from a single patient, results from the primary tumor were taken rather than those from metastases. The resulting PAD dataset is presented in Supplementary Table S1.

### MSI status assignment

Samples with available TMB data but lacking MS data were classified as previously described at length in ref. ^5^. Briefly, based on extensive statistical analysis of TMB distributions, tumors with TMB between 16 and 100 were classified as likely MSI, grouped with known MSI tumors, and designated MT-H; those with TMB < 16 were grouped with known MSS tumors, and designated MT-L. The small number of samples with TMB > 100 were classified as hypermutated MSS tumors (i.e. likely bearing *POLD* or *POLE* mutation), annotated as having a high tumor mutation burden (MSS-htmb). These were removed from subsequent analyses of the MSS/MT-L sub-group, based on known differences in tumor mutational pressures.

To assign MSI status for specimens without pre-calculated TMB (i.e. samples subjected to targeted sequencing using proprietary panel testing for a limited number of genes, e.g. oncopanels), we first determined which of the available parameters (CNA fraction, presence of the mutations in MMR genes, and mutation count) would best partition TCGA COADREAD data into MSS and MSI-H subsets. For this step, we used the R package “party”^61^ with minimum criteria of 0.999 (*p*-value 0.001). Parameters identified in this step were further used to predict the MSI status for each oncopanel. To assess the classification performance of our approach, we used two validation sets for tumors with known MSS and MSI-H status and TMB from ref. ^62^ (Supplementary Table S1, oncopanels IMPACT341 (202 samples) and IMPACT410 (888 samples)), and achieved high classification performance (AUC-ROC of 1 and 0.94, correspondingly).

### Statistical analysis

Data were analyzed in R version 4.0.3 using RStudio. Relationships between predicted and observed mutational profiles were assessed using modified chi-square test. Lipid phosphatase activity (LPA), abundance, and allele fraction (AF) profiles were compared using a Kolmogorov–Smirnov (KS) test. Co-occurrence or mutual exclusion of mutations was calculated using a Fisher’s exact test. To account for multiple comparisons of various types, we have lowered the threshold for statistical significance tenfold, to 0.005. Single-residue and 3D hotspots were identified using previously described methods^8^.

To improve identification of 3D hotspots, the ESMfold model was generated using the Web resource (https://esmatlas.com/) and AlphaFold2 models were generated using ColabFold v.1.2.0^63^. Specifically, the AlphaFold2-ptm model type with 24 recycles was used with the MMSeq2 (Uniref + Environmental) and unpaired + paired mode, based on amino acid residues 1–354 of PTEN. Models were subsequently refined using OpenMM and Amber Force Fields^64^. The local Distance Difference Test (lDDT) was used to assess local model quality, and 5 top ranked models were selected. Two residues with any pair of atoms within 5 Å were considered in contact, with distances calculated using the Residue Interaction Network Generator (RING) (ref. ^65^, https://ring.biocomputingup.it/submit). 3D hotspots were determined as previously described^5^. To determine amino acids at the interface between two PTEN molecules, we identified all pairs of residues in contact between two chains in 5BUG [https://www.rcsb.org/structure/5BUG], 5BZX [https://www.rcsb.org/structure/5BZX]), and 5BZZ [https://www.rcsb.org/structure/5BZZ]). We considered the contact between the two residues as true if proximity was reproducible in at least 4 out of 6 chain-chain interacting pairs. PyMol (ref. ^66^, https://pymol.org/2/) was used for the model alignment, as well as for the visualization of relevant residues on the corresponding structures.

### Mutational signatures

Information on the mutational signatures prevalent in colorectal cancer was downloaded from the COSMIC database (version 3.1); https://cancer.sanger.ac.uk/cosmic/signatures). Compatibility of the detected mutation with a given signature was determined by matching observed mutations to the most frequent nucleotide base changes, together with its trinucleotide context, in each signature.

### Assessment of PTEN variant functionality

Estimates of lipid phosphatase activity and protein abundance were based on data reported in ref. ^18^. Prediction of the impact of in frame deletions on protein function were done using MutPred2^20^, and annotations in clinical databases were retrieved from ref. ^5^.

### Reporting summary

Further information on research design is available in the Nature Research Reporting Summary linked to this article.

### Supplementary information

Supplementary Data

Supplemental Figures

REPORTING SUMMARY

## Data Availability

The sequencing data provided by Foundation Medicine in this study are derived from clinical samples. All consented data supporting the findings of this study that can be released are provided within the article and its supplementary files. Due to HIPAA requirements, we are not authorized to share underlying sequence data or individualized patient genomic data, which contain potentially identifying or sensitive patient information. Foundation Medicine, Inc. is committed to collaborative data analysis, and it has well-established and widely utilized mechanisms by which investigators who have signed a data transfer agreement can query its core genomic database of >600,000 deidentified sequenced cancers to obtain aggregated datasets. For more information and mechanisms of access to the Foundation Medicine, Inc. data in this study, please contact the corresponding authors or the Foundation Medicine, Inc. Data Governance Council at data.governance.council@foundationmedicine.com. Publicly available web resources used in this paper are listed here: The cBioPortal for Cancer Genomics, https://www.cbioportal.org ; AACR Project GENIE, https://genie.cbioportal.org; the Catalogue Of Somatic Mutations In Cancer, https://cancer.sanger.ac.uk/cosmic; the Surveillance, Epidemiology, and End Results (SEER) Program, https://seer.cancer.gov; ICGC, https://dcc.icgc.org. PyMol files for the visualization of hotspots on the PTEN structure (used in Fig. 1d, e) are available in the corresponding folders at Github (https://github.com/NDeeSeee/PTEN_CRC_2023/paper).

## Code availability

The code used for the data analysis in this study is deposited at Zenodo (https://zenodo.org/record/8423389).
