# Supplementary material for: Source, co-occurrence, and prognostic value of PTEN mutations or loss in colorectal cancer
Source: NPJ Genom Med. 2023 Nov 24;8:40. doi: 10.1038/s41525-023-00384-7 (PMC10674024; doi:10.1038/s41525-023-00384-7)
Supplement: Supplementary file 2 — Supplemental Figures [file 41525_2023_384_MOESM2_ESM.pdf]

a

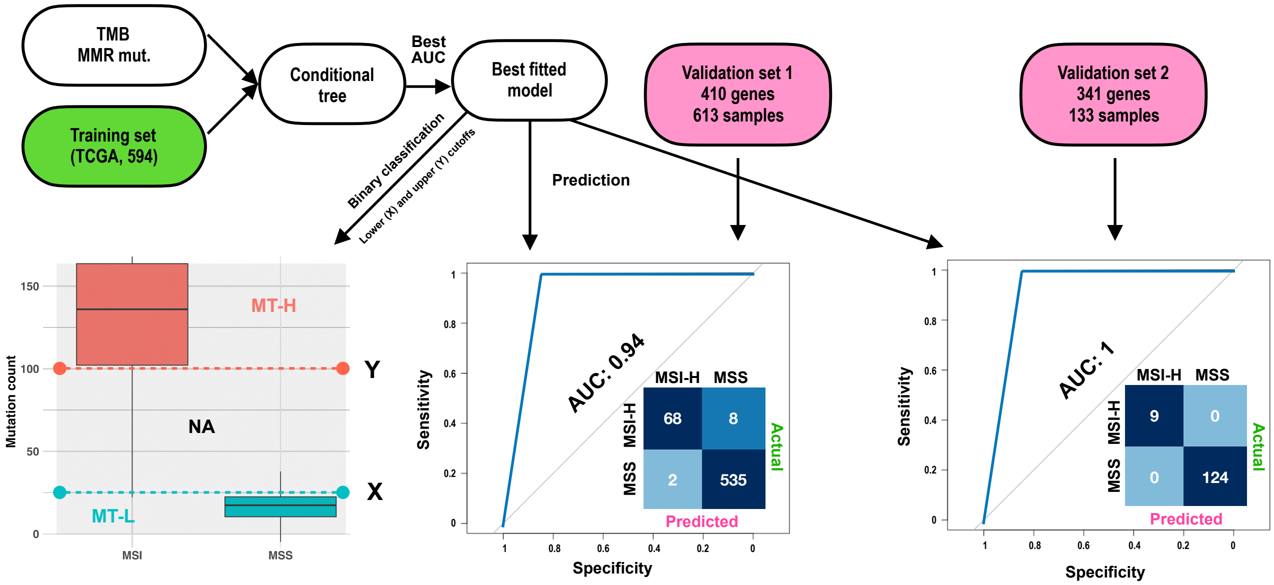

b

DB	#	Study	#	MS measured	MS imputed	SITE	OS
GENIE	10856	GENIE V.13	10856	-	6847	10856	822
cBioPortal	6830	MSKCC studies merged	4179	4069	-	4179	3656
		pan_origimed_2020	1225	-	1208	1225	-
		coadread_dfci_2016	619	531	88	619	-
		coadread_tcga_pan_can_atlas_2018	594	451	143	594	591
		coad_cptac_2019	110	105	-	109	102
		coadread_genentech	74	74	-	74	-
		coad_caseccc_2015	29	29	-	21	-
Papers	672	MMCC (Nat Commun 2016)	487	487	-	487	-
		DFCI (Nat Genet 2014)	185	168	-	185	-
ICGC	321	COCA-CN	321	-	309	-	321
Sum	18679		18679	5914	8595	18349	5492

c

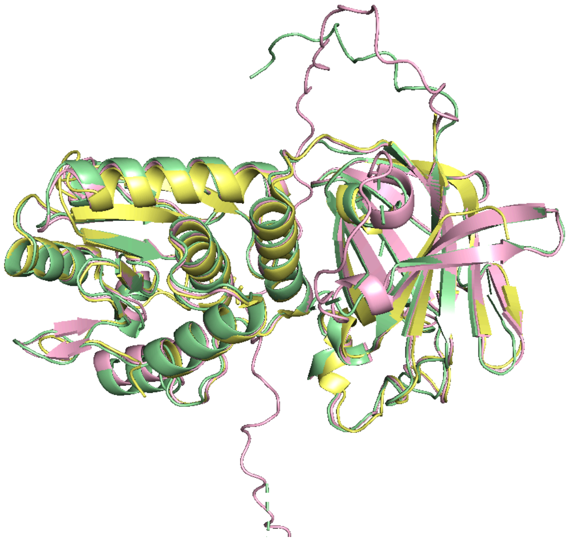

d

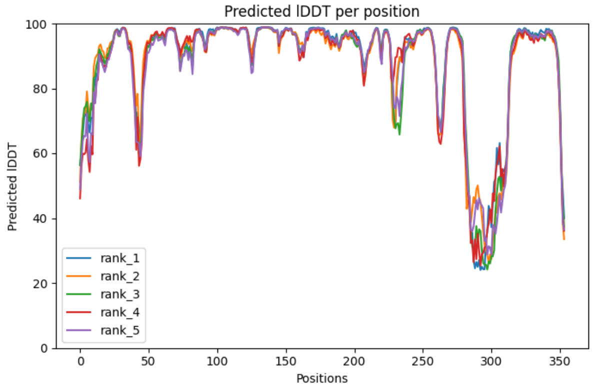

Fig. S1, Serebriiskii et al.

Suppl Figure S1. Definition of CRC datasets, and 3D modeling of PTEN structures. A. Algorithm used to assign MS status to the samples without explicit classification by data provider. At the first step, we used a conditional tree algorithm to determine which of the parameters available for a given oncopanel (CNA fraction, presence of the mutations in MMR genes, and mutation count) would best partition TCGA COADREAD data into MSS and MSI-H subsets. Subsequently, we used these parameters to separate samples analyzed within that particular oncopanel into MSS and MSI-H subsets (e.g., as illustrated by the lower left panel, we used two cutoff scores defining higher bound of MSS and lower bound of MSI-high subsets, leaving the samples between these bounds as undefined). To assess the overall efficiency of our approach, we used two different validation sets with known MSS/MSI-H composition, and achieved high classification performance for both (AUC-ROC of 1 and 0.94). **B**. Overall characterization of PAD dataset**.** “MSK studies merged” include data from the studies coadread_mskcc, crc_msk, msk_impact, rectal_msk, crc_apc_impact, msk_met, bowel_colitis_msk, coadread_mskresistance, and crc_dd. “Papers” refer to data extracted from the supplementary tables of (1,2). Column names denote the following: “MS original”, MS status provided in the original data; “MS imputed “, MS status imputed in this work as described in Supplementary Materials and Methods; “Site”, the availability of any anatomical identifier more detailed than “colorectal” (i.e., “cecum” or “descending colon”); “OS”, survival data available. **C**. PTEN structures predicted by ESMfold (https://esmatlas.com/)(pink) and AlphaFold (https://alphafold.ebi.ac.uk/entry/P60484) (yellow) superimposed on experimentally determined crystal structure 1D5R https://www.rcsb.org/structure/1d5r (green). The predicted models share high structural similarity with PTEN structures available in PDB. **D.** The local Distance Difference Test (lDDT) (3) scores for the 5 best AlphaFold2 models used in predicting 3D hotspots indicate the high

a

```
        10        20        30        40        50        60        70        80        90
ATGACAGCCATCATCAAAGAGATCGTTAGCAGAAACAAAAGGAGATATCAAGAGGATGGATTCGACTTAGACTTGACCTATATTTATCCAAACATTATTG  100
 M  T  A  I  I  K  E  I  V  S  R  N  K  R  R  Y  Q  E  D  G  F  D  L  D  L  T  Y  I  Y  P  N  I  I  A  34
                                                                                          delATT 12/6

CTATGGGATTTCCTGCAGAAAGACTTGAAGGCGTATACAGGAACAATATTGATGATGTAGTAAGGTTTTTGGATTCAAAGCATAAAAACCATTACAAGAT  200
 M  G  F  P  A  E  R  L  E  G  V  Y  R  N  N  I  D  D  V  V  R  F  L  D  S  K  H  K  N  H  Y  K  I  67
                                                           delT 2/4          delA 0/13
                                                           insT 7/5          insA 2/0

ATACAATCTTTGTGCTGAAAGACATTATGACACCGCCAAATTTAATTGCAGAGTTGCACAATATCCTTTTGAAGACCATAACCCACCACAGCTAGAACTT  300
 Y  N  L  C  A  E  R  H  Y  D  T  A  K  F  N  C  R  V  A  Q  Y  P  F  E  D  H  N  P  P  Q  L  E  L  100
                     delTAT 1/4                                   delT 0/8
                                                                  insT 1/1

ATCAAACCCTTTTGTGAAGATCTTGACCAATGGCTAAGTGAAGATGACAATCATGTTGCAGCAATTCACTGTAAAGCTGGAAAGGGACGAACTGGTGTAA  400
 I  K  P  F  C  E  D  L  D  Q  W  L  S  E  D  D  N  H  V  A  A  I  H  C  K  A  G  K  G  R  T  G  V  M  134
        delT 0/3

TGATATGTGCATATTTATTACATCGGGGCAAATTTTTAAAGGCACAAGAGGCCCTAGATTTCTATGGGGAAGTAAGGACCAGAGACAAAAAGGGAGTAAC  500
 I  C  A  Y  L  L  H  R  G  K  F  L  K  A  Q  E  A  L  D  F  Y  G  E  V  R  T  R  D  K  K  G  V  T  167
                              delT 2/2                          delG 1/3           delA 0/9
                              insT 2/2                          insG 4/18          insA 5/0

TATTCCCAGTCAGAGGCGCTATGTGTATTATTATAGCTACCTGTTAAAGAATCATCTGGATTATAGACCAGTGGCACTGTTGTTTCACAAGATGATGTTT  600
 I  P  S  Q  R  R  Y  V  Y  Y  Y  S  Y  L  L  K  N  H  L  D  Y  R  P  V  A  L  L  F  H  K  M  M  F  200

GAAACTATTCCAATGTTCAGTGGCGGAACTTGCAATCCTCAGTTTGTGGTCTGCCAGCTAAAGGTGAAGATATATTCCTCCAATTCAGGACCCACACGAC  700
E  T  I  P  M  F  S  G  G  T  C  N  P  Q  F  V  V  C  Q  L  K  V  K  I  Y  S  S  N  S  G  P  T  R  R  234

GGGAAGACAAGTTCATGTACTTTGAGTTCCCTCAGCCGTTACCTGTGTGTGGTGATATCAAAGTAGAGTTCTTCCACAAACAGAACAAGATGCTAAAAAA  800
   E  D  K  F  M  Y  F  E  F  P  Q  P  L  P  V  C  G  D  I  K  V  E  F  F  H  K  Q  N  K  M  L  K  K  267
                                                                                        delA 2/130
                                                                                        insA 4/19

GGACAAAATGTTTCACTTTTGGGTAAATACATTCTTCATACCAGGACCAGAGGAAACCTCAGAAAAAGTAGAAAATGGAAGTCTATGTGATCAAGAAATC  900
 D  K  M  F  H  F  W  V  N  T  F  F  I  P  G  P  E  E  T  S  E  K  V  E  N  G  S  L  C  D  Q  E  I  300
              delT  0/5                                      delA 2/10
                                                             insA 3/2

GATAGCATTTGCAGTATAGAGCGTGCAGATAATGACAAGGAATATCTAGTACTTACTTTAACAAAAAATGATCTTGACAAAGCAAATAAAGACAAAGCCA  1000
D  S  I  C  S  I  E  R  A  D  N  D  K  E  Y  L  V  L  T  L  T  K  N  D  L  D  K  A  N  K  D  K  A  N  334
                                            delACTT 49/7   delA 3/61
                                                           insA 15/11

ACCGATACTTTTCTCCAAATTTTAAGGTGAAGCTGTACTTCACAAAAACAGTAGAGGAGCCGTCAAATCCAGAGGCTAGCAGTTCAACTTCTGTAACACC  1100
  R  Y  F  S  P  N  F  K  V  K  L  Y  F  T  K  T  V  E  E  P  S  N  P  E  A  S  S  S  T  S  V  T  P  367
                                      insA 0/2

AGATGTTAGTGACAATGAACCTGATCATTATAGATATTCTGACACCACTGACTCTGATCCAGAGAATGAACCTTTTGATGAAGATCAGCATACACAAATT  1200
 D  V  S  D  N  E  P  D  H  Y  R  Y  S  D  T  T  D  S  D  P  E  N  E  P  F  D  E  D  Q  H  T  Q  I  400

ACAAAAGTCTGA  1300
T  K  V  *
```

b

Category	poly-nt runs	ins	del	ins:del ratio	Comment	significance
MT-H	Y	39	248	0.16	deletions much more frequent	< 2.2e-16
MT-L	Y	44	16	2.75	insertions more frequent	0.00049
MT-H	N	8	4	2	insertions more frequent	ns
MT-L	N	49	33	1.48	insertions more frequent	ns

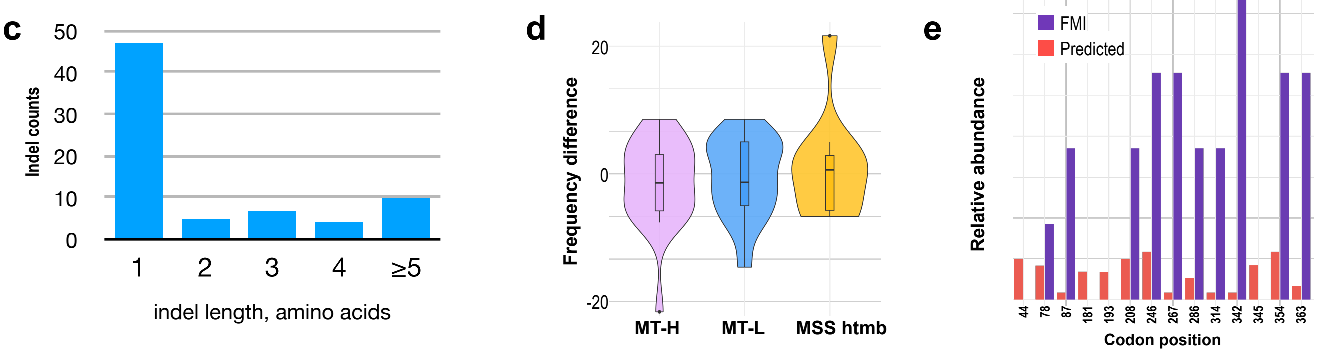

Fig. S2, Serebriiskii et al.

Supplementary Figure 2. Characteristics of nucleotide signatures associated with PTEN hotspot mutations. **A**. CG dinucleotides, for which spontaneous or enzymatic deamination of 5-methylcytosine to thymine generates mutational pattern described by SBS1 signature, are highlighted in yellow. Polynucleotide runs, where DNA polymerase slippage may occur more frequently (ID1/ID2/ID5/ID7 signatures), are shown in orange and annotated with the counts of insertions and deletions in MT-L vs MT-H subsets of FMI data. Two AAAAAA stretches, marked in black and red, differ significantly in observed mutation frequencies in MT-H subset. **B**. Ratio of insertions to deletions occurring in MT-L versus MT-H cohorts is significantly different within polynucleotide runs (Y), but not outside them (N). p-values calculated by the prop.test function in R (4). **C.** Characterization of *PTEN* inframe indels. Of 74 inframe indels in the FMI *PTEN* dataset, four are recurrent, including **Y76del** (VUS ClinVar SCV000596624), **M134del** (VUS ClinVar SCV000692011), and **Y178del** (VUS ClinVar VCV000135910.1). The hotspot **I33del** is reported to be a likely pathogenic mutation (ClinVar VCV000449088.5). It has been found as germline mutation in *PTEN* Hamartoma Tumor syndrome and classified using modified ACMG criteria (5-7). This mutation results from a loss of a microhomology repeat located at microsatellite region, and is attributed to the ID2 signature (a mismatch repair defect). **D.** Distribution of codon use changes for synonymous *PTEN* mutations in MT-L, MT-H, and MSS-htmb cohorts. For each synonymous mutation, the difference in codon usage frequency is calculated versus the WT codon in *H. sapiens*. A significant shift in the distribution towards negative values would have indicated that mutated codons are rarer than the wild type codons, potentially resulting in lower protein abundance due to effects such as reduced translation rates, (8). No such shift is evident, indicating that synonymous mutations are not likely to result in the lower protein levels due to effects on translation (although it is possible they affect additional processes such as interaction with ncRNAs, mRNA stability, or splicing regulation). **E**. Silent mutations with statistically significant differences between predicted and real frequencies. Numbers reflect protein codons affected (not nucleotide

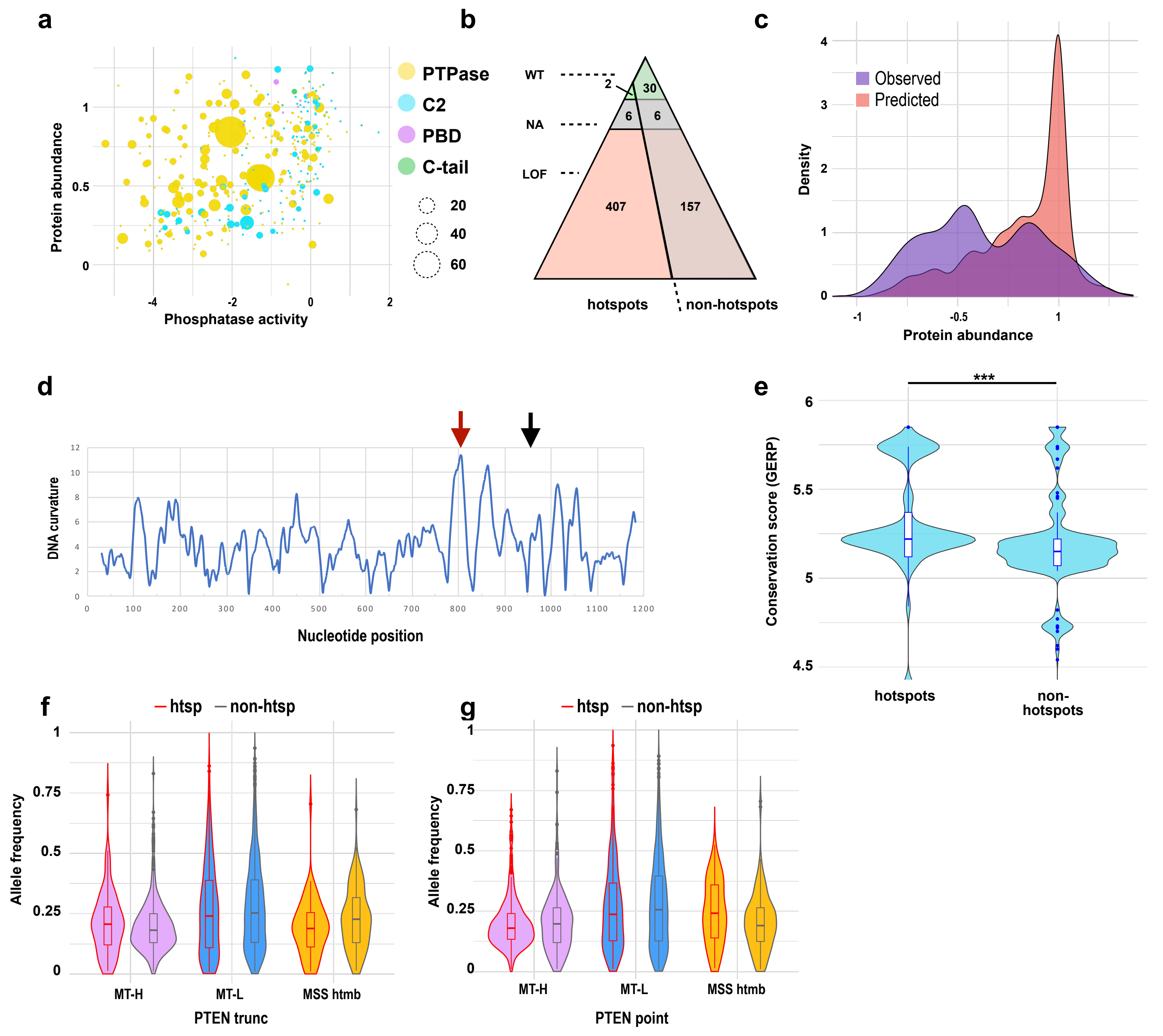

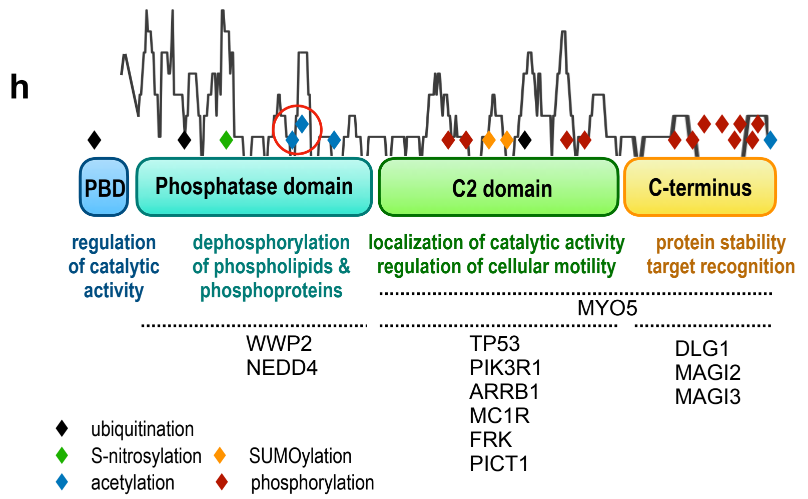

PTEN PTMs

codon	AA	Modification	codon	AA	Modification
13	K	ubiquitination	321	T	phosphorylation
63	K	ubiquitination	336	Y	phosphorylation
83	C	S-nitrosylation	366	T	phosphorylation
125	K	acetylation	370	S	phosphorylation
128	K	acetylation	380	S	phosphorylation
163	K	acetylation	382	T	phosphorylation
229	S	phosphorylation	383	T	phosphorylation
240	Y	phosphorylation	385	S	phosphorylation
254	K	SUMOylation	398	T	phosphorylation
266	K	SUMOylation	401	T	phosphorylation
289	K	ubiquitination	402	K	acetylation

Fig. S3, Serebriiskii et al.

Supplementary figure 3. Structure-function relations of PTEN mutations in the context of DNA curvature, conformation, and mutation allele frequency. **A**. Extended analysis of full *PTEN* mutation set by LPA and abundance scores for mutations in FMI CRC set, color by *PTEN* domains. For each non-truncating mutation present in the FMI CRC dataset, values for protein abundance and lipid phosphatase activity (LPA) (9) were plotted on the corresponding axes. Bubble size is proportional to the count of the mutations in the analyzed set, and color indicates the *PTEN* domain where the mutation occurs. **B**. For missense mutations in the MT-H dataset, analysis applying cutoffs reported by (9) for significant impairment of protein abundance and lipid phosphatase activity (LPA). Prediction of the impact of inframe deletions on protein function were done using MutPred2. After further stratification by annotation in the clinical databases (CKB, OncoKB, Clinvar; see Methods for details), mutations (both hotspot and non-hotspot) were assigned as functionally wild type (WT) or loss of function (LoF); NA, not available, signified insufficient data for prediction. LoF mutations are more frequent among the hotspots (p-value < 2.2e-16). **C.** Comparison of the predicted (red) vs observed (blue) protein abundance profile for *PTEN* missense mutations in the MT-L subset. For each predicted or observed mutations, the corresponding LPA values were retrieved from (9), see Supp Table S8. **D.** DNA curvature along the *PTEN* open reading frame was determined using the Bend-It server (10) (http//pongor.itk.ppke.hu/dna/bend_it.html). Arrows indicating positions of the two AAAAAA polynucleotide runs, with higher DNA curvature at amino acid 800 than 960**.** There are approximately twice as many mutations at position 800 as at position 960, which may be explained by studies linking DNA curvature to polymerase slippage during replication (11). **E.** Degree of DNA conservation in mutations targeted by hotspot versus non-hotspot mutations (missense and inframe indels) indicates hotspot mutations do not target more highly conserved nt. ***, p=1.7E-7. Y-axis, Genomic Evolutionary Rate Profiling (GERP) score (12), with higher scores indicating higher evolutionary constraint. **F-G.** *PTEN* hotspots (versus not hotspots) by AF by point (missense, inframe indel) (**F**) versus truncating (**G**) mutations, in the MT-L, MT-H, and MSS-htmb subsets. No statistically significant differences were found. Abbreviations: "htsp", mutations in the category of hotspots as defined in Supp. Table S2; "non-htsp", cumulatively all other non-synonymous mutations. **H.** Distribution of WT-like *PTEN* mutations along the *PTEN* protein sequence, in relation to the sites of post-translational modifications (PTMs) and approximate binding sites of key PTEN interacting proteins. Density of the *PTEN* WT-like mutations (calculated using a sliding window of 9 aa) is shown as black curved line, with the peaks corresponding to a higher mutation count in the indicated region of the protein. PTMs are shown as colored rhomboid symbols, while representative validated proteins, for which the location of the interaction with *PTEN* is known, are shown beneath the protein cartoon (TP53, (13); ARRB1, (14); PIK3R1, (15); MYO5, (16); DLG1, (17); all others, as reviewed in (18,19)**.** No statistically significant enrichment of wt-like *PTEN* mutations in the vicinity (+/- 2 aa) of the PTM sites on *PTEN* was found, and the fraction of *PTEN* wt-like mutations was similar

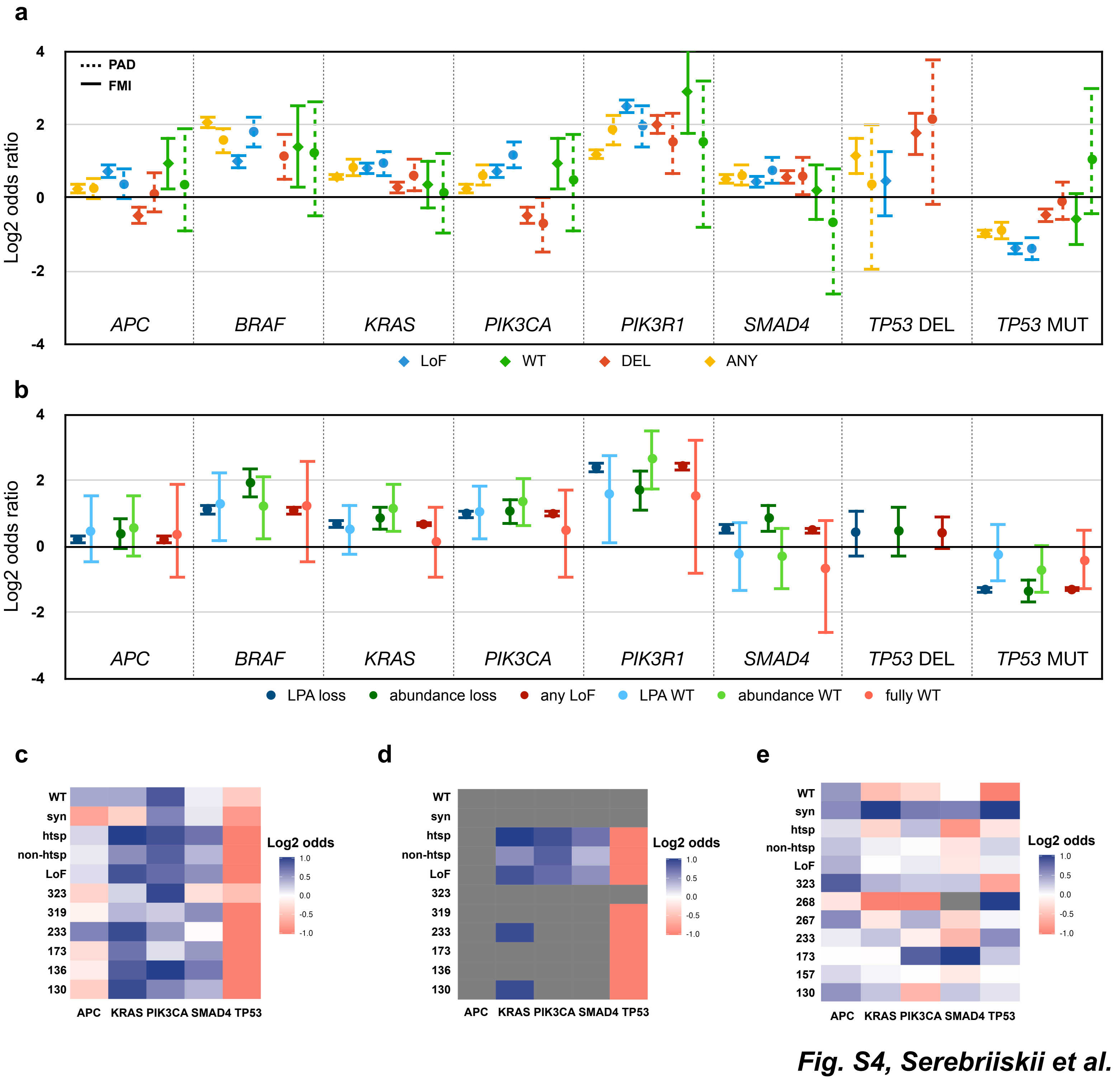

Fig. S4, Serebriiskii et al.

Suppl. Figure 4. Co-occurrence and mutual exclusion of PTEN alterations with common CRC driver mutations. A. Co-occurrence of *PTEN* LoF or WT-like mutations with alterations in *APC*, *TP53*, *PIK3CA*, *SMAD4*, or *KRAS*, calculated separately for the FMI and PAD datasets of MT-L CRC. **B.** Co-occurrence of *PTEN* mutations with alterations in *APC*, *TP53*, *PIK3CA*, *SMAD4*, or *KRAS*, calculated for the MT-L subset of combined set of FMI and PAD data; separately for the loss of LPA, abundance, or consensus LoF as defined in Material and Methods. **C-E**. Co-occurrence of individual *PTEN* hotspots, versus all other hotspots, or versus LoF, synonymous, or WT-like mutations. Abbreviations: "htsp", mutations in the category of subset-specific hotspots, excluding those in codons listed below in the panel; "non-htsp", cumulatively all other non-synonymous mutations; "syn", synonymous. **C.** Patterns of co-occurrence and mutual exclusion for mutations in the genes indicated, in the merged FMI and PAD datasets MT-L cohort. Darker shades of blue color indicate greater co-occurrence, and shades of red color indicate mutual exclusion. **D.** Patterns of co-occurrence and mutual exclusion as in **C**, with the gray color indicating non-significant results (p-values >0.005). **E.** Patterns of co-occurrence and mutual exclusion as in **C**, calculated for the MT-H subset of the merged FMI and PAD datasets. No individual results satisfying the criteria of statistical significance (p-values <0.005) were found.

References for Supplementary Legends.

1. Schell MJ, Yang M, Teer JK, Lo FY, Madan A, Coppola D*, et al.* A multigene mutation classification of 468 colorectal cancers reveals a prognostic role for APC. *Nat Commun* 2016;**7**:11743 doi 10.1038/ncomms11743.
2. Giannakis M, Hodis E, Jasmine Mu X, Yamauchi M, Rosenbluh J, Cibulskis K*, et al.* RNF43 is frequently mutated in colorectal and endometrial cancers. *Nat Genet* 2014;**46**(12):1264-6 doi 10.1038/ng.3127.
3. Mariani V, Biasini M, Barbato A, Schwede T. lDDT: a local superposition-free score for comparing protein structures and models using distance difference tests. *Bioinformatics* 2013;**29**(21):2722-8 doi 10.1093/bioinformatics/btt473.
4. Newcombe RG. Interval estimation for the difference between independent proportions: comparison of eleven methods. *Stat Med* 1998;**17**(8):873-90 doi 10.1002/(sici)1097-0258(19980430)17:8<873::aid-sim779>3.0.co;2-i.
5. Mester JL, Ghosh R, Pesaran T, Huether R, Karam R, Hruska KS*, et al.* Gene-specific criteria for PTEN variant curation: Recommendations from the ClinGen PTEN Expert Panel. *Hum Mutat* 2018;**39**(11):1581-92 doi 10.1002/humu.23636.
6. Mighell TL, Evans-Dutson S, O'Roak BJ. A Saturation Mutagenesis Approach to Understanding PTEN Lipid Phosphatase Activity and Genotype-Phenotype Relationships. *Am J Hum Genet* 2018;**102**(5):943-55 doi 10.1016/j.ajhg.2018.03.018.
7. Lek M, Karczewski KJ, Minikel EV, Samocha KE, Banks E, Fennell T*, et al.* Analysis of protein-coding genetic variation in 60,706 humans. *Nature* 2016;**536**(7616):285-91 doi 10.1038/nature19057.
8. Ran X, Xiao J, Cheng F, Wang T, Teng H, Sun Z. Pan-cancer analyses of synonymous mutations based on tissue-specific codon optimality. *Comput Struct Biotechnol J* 2022;**20**:3567-80 doi 10.1016/j.csbj.2022.07.005.
9. Mighell TL, Thacker S, Fombonne E, Eng C, O'Roak BJ. An Integrated Deep-Mutational-Scanning Approach Provides Clinical Insights on PTEN Genotype-Phenotype Relationships. *Am J Hum Genet* 2020;**106**(6):818-29 doi 10.1016/j.ajhg.2020.04.014.
10. Vlahovicek K, Kajan L, Pongor S. DNA analysis servers: plot.it, bend.it, model.it and IS. *Nucleic Acids Res* 2003;**31**(13):3686-7 doi 10.1093/nar/gkg559.
11. Zhang H, Li D, Zhao X, Pan S, Wu X, Peng S*, et al.* Relatively semi-conservative replication and a folded slippage model for short tandem repeats. *BMC Genomics* 2020;**21**(1):563 doi 10.1186/s12864-020-06949-5.
12. Cooper GM, Stone EA, Asimenos G, Program NCS, Green ED, Batzoglou S*, et al.* Distribution and intensity of constraint in mammalian genomic sequence. *Genome research* 2005;**15**(7):901-13 doi 10.1101/gr.3577405.
13. Freeman DJ, Li AG, Wei G, Li HH, Kertesz N, Lesche R*, et al.* PTEN tumor suppressor regulates p53 protein levels and activity through phosphatase-dependent and -independent mechanisms. *Cancer Cell* 2003;**3**(2):117-30 doi 10.1016/s1535-6108(03)00021-7.
14. Lima-Fernandes E, Enslen H, Camand E, Kotelevets L, Boularan C, Achour L*, et al.* Distinct functional outputs of PTEN signalling are controlled by dynamic association with beta-arrestins. *The EMBO journal* 2011;**30**(13):2557-68 doi 10.1038/emboj.2011.178.
15. Marshall JDS, Mellor P, Ruan X, Whitecross DE, Moore SA, Anderson DH. Insight into the PTEN - p85alpha interaction and lipid binding properties of the p85alpha BH domain. *Oncotarget* 2018;**9**(97):36975-92 doi 10.18632/oncotarget.26432.
16. van Diepen MT, Parsons M, Downes CP, Leslie NR, Hindges R, Eickholt BJ. MyosinV controls PTEN function and neuronal cell size. *Nat Cell Biol* 2009;**11**(10):1191-6 doi 10.1038/ncb1961.
17. Valiente M, Andres-Pons A, Gomar B, Torres J, Gil A, Tapparel C*, et al.* Binding of PTEN to specific PDZ domains contributes to PTEN protein stability and phosphorylation by microtubule-associated serine/threonine kinases. *J Biol Chem* 2005;**280**(32):28936-43 doi 10.1074/jbc.M504761200.
18. Smith SL, Pitt AR, Spickett CM. Approaches to Investigating the Protein Interactome of PTEN. *J Proteome Res* 2021;**20**(1):60-77 doi 10.1021/acs.jproteome.0c00570.
19. Lee YR, Chen M, Pandolfi PP. The functions and regulation of the PTEN tumour suppressor: new modes and prospects. *Nat Rev Mol Cell Biol* 2018;**19**(9):547-62 doi 10.1038/s41580-018-0015-0.

Supplementary Methods.

Public Available Data (PAD) curation: Data was collected from the cBioPortal for Cancer Genomics, https://www.cbioportal.org; AACR Project GENIE, https://genie.cbioportal.org (1); the Catalogue Of Somatic Mutations In Cancer (COSMIC), https://cancer.sanger.ac.uk/cosmic (2); ICGC, https://dcc.icgc.org (3); and from data included in the supplementary tables of (4,5). To remove redundancy of the collected data, we implemented the following prioritization rules: 1) All GENIE v.13 samples which overlapped with the studies in cBioPortal were removed. 2) For samples with outcomes progressively described in multiple publications, the studies reported in cBioPortal as of 01.01.2023 were used to establish survival data. 3) For samples without microsatellite (MS) status, but with measures of MS instability predicted by MSI prediction algorithms (e.g., SENSOR (6) or MANTIS (7) scores), we assigned MS status according to the cutoffs used in the corresponding studies (3.5 and 0.4, respectively). 4) In cases where multiple specimens were collected from a single patient, results from the primary tumor were taken rather than those from metastases. Data describing PAD samples are presented in Supp Table S1.

MSI status assignment: Samples with available TMB data but lacking MS data were classified as previously described (8). Briefly, tumors with TMB between 16 and 100 were classified as likely MSI, grouped with known MSI tumors, and designated MT-H; those with TMB<16 were grouped with known MSS tumors, and designated MT-L. The small number of samples with TMB> 100 were classified as hypermutated MSS tumors, bearing a high tumor mutation burden (MSS-htmb), and removed from subsequent analyses of the MSS/MT-L sub-group).

To assign MSI status for specimens without pre-calculated TMB (i.e. samples subjected to targeted sequencing using proprietary panel testing for a limited number of genes, e.g. oncopanels), we first determined which of the available parameters (CNA fraction, presence of the mutations in MMR genes, and mutation count) would best partition TCGA COADREAD data into MSS and MT-H subsets. For this step, we used the R package "party" (9) with minimum criteria of 0.999 (p-value 0.001). Parameters identified in this step were further used to predict the MSI status for each oncopanel. To assess the classification performance of our approach, we used two validation sets for tumors with known MSS and MT-H status and TMB from (10) (Supp Table 1, oncopanels IMPACT341 (202 samples) and IMPACT410 (888 samples)), and achieved high classification performance (AUC-ROC of 1 and 0.94, correspondingly).

Using predictions of PTEN protein structure to improve identification of 3D hotspots: The ESMfold model was generated using the Web resource (https://esmatlas.com/) and AlphaFold2 models were generated using ColabFold v.1.2.0 (11). Specifically, the AlphaFold2-ptm model type with 24 recycles was used with the MMSeq2 (Uniref+Environmental) and unpaired+paired mode, based on amino acid residues 1-354 of PTEN. Models were subsequently refined using OpenMM and Amber Force Fields (12). The local Distance Difference Test (lDDT) was used to assess local model quality, and 5 top ranked models were selected. Two residues with any pair of atoms within 5Å were considered in contact, with distances calculated using the Residue Interaction Network Generator (RING) ((13), https://ring.biocomputingup.it/submit). 3D hotspots were determined as previously described (8). To determine amino acids at the interface between two PTEN molecules, we identified all pairs of residues in contact between two chains in 5BUG [https://www.rcsb.org/structure/5BUG], 5BZX [https://www.rcsb.org/structure/5BZX]), and 5BZZ [https://www.rcsb.org/structure/5BZZ]). We considered the contact between the two residues as true if proximity was reproducible in at least 4 out of 6 chain-chain interacting pairs. PyMol ((14), https://pymol.org/2/) was used for the model alignment, as well as for the visualization of relevant residues on the corresponding structures.

References for Supplementary Methods.

1. Consortium APG. AACR Project GENIE: Powering Precision Medicine through an International Consortium. *Cancer Discov* 2017;**7**(8):818-31 doi 10.1158/2159-8290.CD-17-0151.
2. Forbes SA, Beare D, Boutselakis H, Bamford S, Bindal N, Tate J*, et al.* COSMIC: somatic cancer genetics at high-resolution. *Nucleic Acids Res* 2017;**45**(D1):D777-D83 doi 10.1093/nar/gkw1121.
3. Zhang J, Bajari R, Andric D, Gerthoffert F, Lepsa A, Nahal-Bose H*, et al.* The International Cancer Genome Consortium Data Portal. *Nat Biotechnol* 2019;**37**(4):367-9 doi 10.1038/s41587-019-0055-9.
4. Schell MJ, Yang M, Teer JK, Lo FY, Madan A, Coppola D*, et al.* A multigene mutation classification of 468 colorectal cancers reveals a prognostic role for APC. *Nat Commun* 2016;**7**:11743 doi 10.1038/ncomms11743.
5. Giannakis M, Hodis E, Jasmine Mu X, Yamauchi M, Rosenbluh J, Cibulskis K*, et al.* RNF43 is frequently mutated in colorectal and endometrial cancers. *Nat Genet* 2014;**46**(12):1264-6 doi 10.1038/ng.3127.
6. Niu B, Ye K, Zhang Q, Lu C, Xie M, McLellan MD*, et al.* MSIsensor: microsatellite instability detection using paired tumor-normal sequence data. *Bioinformatics* 2014;**30**(7):1015-6 doi 10.1093/bioinformatics/btt755.
7. Kautto EA, Bonneville R, Miya J, Yu L, Krook MA, Reeser JW*, et al.* Performance evaluation for rapid detection of pan-cancer microsatellite instability with MANTIS. *Oncotarget* 2017;**8**(5):7452-63 doi 10.18632/oncotarget.13918.
8. Serebriiskii IG, Pavlov V, Tricarico R, Andrianov G, Nicolas E, Parker MI*, et al.* Comprehensive characterization of PTEN mutational profile in a series of 34,129 colorectal cancers. *Nat Commun* 2022;**13**(1):1618 doi 10.1038/s41467-022-29227-2.
9. Buri M, Tanadini LG, Hothorn T, Curt A. Unbiased Recursive Partitioning Enables Robust and Reliable Outcome Prediction in Acute Spinal Cord Injury. *J Neurotrauma* 2022;**39**(3-4):266-76 doi 10.1089/neu.2020.7407.
10. Yaeger R, Chatila WK, Lipsyc MD, Hechtman JF, Cercek A, Sanchez-Vega F*, et al.* Clinical Sequencing Defines the Genomic Landscape of Metastatic Colorectal Cancer. *Cancer Cell* 2018;**33**(1):125-36 e3 doi 10.1016/j.ccell.2017.12.004.
11. Mirdita M, Schutze K, Moriwaki Y, Heo L, Ovchinnikov S, Steinegger M. ColabFold: making protein folding accessible to all. *Nature methods* 2022;**19**(6):679-82 doi 10.1038/s41592-022-01488-1.
12. Eastman P, Swails J, Chodera JD, McGibbon RT, Zhao Y, Beauchamp KA*, et al.* OpenMM 7: Rapid development of high performance algorithms for molecular dynamics. *PLoS Comput Biol* 2017;**13**(7):e1005659 doi 10.1371/journal.pcbi.1005659.
13. Clementel D, Del Conte A, Monzon AM, Camagni GF, Minervini G, Piovesan D*, et al.* RING 3.0: fast generation of probabilistic residue interaction networks from structural ensembles. *Nucleic Acids Res* 2022;**50**(W1):W651-W6 doi 10.1093/nar/gkac365.
14. DeLano W. The PyMOL User's Manual. DeLano Scientific, San Carlos,CA, USA 2002.
