# Supplementary material for: Source, co-occurrence, and prognostic value of PTEN mutations or loss in colorectal cancer
Source: NPJ Genom Med. 2023 Nov 24;8:40. doi: 10.1038/s41525-023-00384-7 (PMC10674024; doi:10.1038/s41525-023-00384-7)
Supplement: Supplementary file 3 — REPORTING SUMMARY [file 41525_2023_384_MOESM3_ESM.pdf]

nature research

Corresponding author(s): Erica A. Golemis, Ilya G. Serebriiskii

Reporting Summary

Nature Research wishes to improve the reproducibility of the work that we publish. This form provides structure for consistency and transparency in reporting. For further information on Nature Research policies, see Authors & Referees and the Editorial Policy Checklist.

Statistical parameters

When statistical analyses are reported, confirm that the following items are present in the relevant location (e.g. figure legend, table legend, main text, or Methods section).

n/a	Confirmed	
☐	☒	The exact sample size (n) for each experimental group/condition, given as a discrete number and unit of measurement
☐	☒	An indication of whether measurements were taken from distinct samples or whether the same sample was measured repeatedly
☐	☒	The statistical test(s) used AND whether they are one- or two-sided<br>*Only common tests should be described solely by name; describe more complex techniques in the Methods section.*
☐	☒	A description of all covariates tested
☐	☒	A description of any assumptions or corrections, such as tests of normality and adjustment for multiple comparisons
☐	☒	A full description of the statistics including central tendency (e.g. means) or other basic estimates (e.g. regression coefficient) AND variation (e.g. standard deviation) or associated estimates of uncertainty (e.g. confidence intervals)
☐	☒	For null hypothesis testing, the test statistic (e.g. F, t, r) with confidence intervals, effect sizes, degrees of freedom and P value noted<br>*Give P values as exact values whenever suitable.*
☒	☐	For Bayesian analysis, information on the choice of priors and Markov chain Monte Carlo settings
☒	☐	For hierarchical and complex designs, identification of the appropriate level for tests and full reporting of outcomes
☒	☐	Estimates of effect sizes (e.g. Cohen's d, Pearson's r), indicating how they were calculated
☐	☒	Clearly defined error bars<br>*State explicitly what error bars represent (e.g. SD, SE, CI)*

Our web collection on statistics for biologists may be useful.

Software and code

Policy information about availability of computer code

Data collection	Data collection and annotation were described in detail in PMID:24142049 (Frampton et al 2013) in a proprietary data collection pipeline developed by FMI. Sequence data were processed using a customized analysis pipeline designed to accurately detect multiple classes of genomic alterations. All testing was done in a CLIA-certified, CAP-accredited laboratory.Data collection and annotation were described in detail in PMID:24142049 (Frampton et al 2013) in a proprietary data collection pipeline developed by FMI. Sequence data were processed using a customized analysis pipeline designed to accurately detect multiple classes of genomic alterations. All testing was done in a CLIA-certified, CAP-accredited laboratory.Additional data were aggregated from cBioPortal, GENIE, ICGC, and peer-reviewed publications.
Data analysis	Statistical analysis was conducted using R 4.3.1 and RStudio with tidyverse libraries; all custom scripts used in this research are deposited at DOI: 10.5281/zenodo.8408675

For manuscripts utilizing custom algorithms or software that are central to the research but not yet described in published literature, software must be made available to editors/reviewers upon request. We strongly encourage code deposition in a community repository (e.g. GitHub). See the Nature Research guidelines for submitting code & software for further information.

Data

Policy information about availability of data

All manuscripts must include a data availability statement. This statement should provide the following information, where applicable:

- Accession codes, unique identifiers, or web links for publicly available datasets
- A list of figures that have associated raw data
- A description of any restrictions on data availability

The sequencing data provided by Foundation Medicine in this study are derived from clinical samples. All consented data supporting the findings of this study that can be released are provided within the article and its supplementary files. Due to HIPAA requirements, we are not authorized to share underlying sequence data or individualized patient genomic data, which contain potentially identifying or sensitive patient information. Foundation Medicine, Inc. is committed to collaborative data analysis, and it has well-established and widely utilized mechanisms by which investigators can query its core genomic database of >600,000 deidentified sequenced cancers to obtain aggregated datasets. For more information and mechanisms of access to the Foundation Medicine, Inc. data in this study, please contact the corresponding authors or the Foundation Medicine, Inc. Data Governance Council at data.governance.council@foundationmedicine.com. You and your institution will be required to sign a data transfer agreement.Web resources used in this paper are listed here: The cBioPortal for Cancer Genomics, https://www.cbioportal.org ; AACR Project GENIE, https://genie.cbioportal.org; the Catalogue Of Somatic Mutations In Cancer, https://cancer.sanger.ac.uk/cosmic; the Surveillance, Epidemiology, and End Results (SEER) Program, https://seer.cancer.gov.

Field-specific reporting

Please select the best fit for your research. If you are not sure, read the appropriate sections before making your selection.

☒ Life sciences ☐ Behavioural & social sciences

For a reference copy of the document with all sections, see nature.com/authors/policies/ReportingSummary-flat.pdf

Life sciences

Study design

All studies must disclose on these points even when the disclosure is negative.

Sample size	The dataset consists of 34,129 CRC samples obtained from Foundation Medicine Inc., supplemented by an additional 18,679 samples from publicly available sources like cBioPortal and GENIE. While the sample size was not pre-calculated, its combined size of 52,808 samples ensures sufficient statistical power, even when subdividing into various subgroups for analysis.
Data exclusions	Data missing some key clinical parameters were excluded from analyses relevant to that specific parameter. Germline mutations were excluded from the analyses, as the focus of this study is solely on somatic mutations.
Replication	All analyses were conducted on genomic data obtained from Foundation Medicine Inc., supplemented by 18,679 publicly available samples from cBioPortal and GENIE. The code used for data preprocessing, analysis, and visualization is publicly available on GitHub, ensuring that the analyzes are fully reproducible. There was no feasibility for performing a replication study based on the additional data, because the dataset used was based on the entire set of tumors sequenced over multiple years by leading genomics research entities - there is no way to accrue a similar cohort without waiting several years.
Randomization	Randomization is not applicable to this study as it is a computational analysis of pre-existing genomic datasets. The allocation of samples into subgroups for analysis was based on specific genomic features or clinical metadata rather than random assignment.
Blinding	Blinding was not applicable to this study, as it is a computational analysis of pre-existing genomic data. There were no experimental groups where blinding could be implemented to prevent bias. The focus of the research is on statistical and computational methods applied to large genomic datasets, and thus blinding is not a relevant consideration in this context.

Materials & experimental systems

Policy information about availability of materials

n/a	Involved in the study
☒	☐ Unique materials
☒	☐ Antibodies
☒	☐ Eukaryotic cell lines
☒	☐ Research animals
☒	☐ Human research participants

Method-specific reporting

n/a	Involved in the study
☒	☐ ChIP-seq
☒	☐ Flow cytometry
☒	☐ Magnetic resonance imaging
